# Identification of genes regulated by trait sensitivity to negative feedback and prolonged alcohol consumption in rats

**DOI:** 10.1007/s43440-023-00563-4

**Published:** 2024-01-03

**Authors:** Agata Cieslik-Starkiewicz, Karolina Noworyta, Joanna Solich, Agata Korlatowicz, Agata Faron-Górecka, Rafal Rygula

**Affiliations:** 1 Affective Cognitive Neuroscience Laboratory, Department of Pharmacology, Maj Institute of Pharmacology Polish Academy of Sciences, 12 Smętna Street, 31-343 Kraków, Poland; 2 Biochemical Pharmacology Laboratory, Department of Pharmacology, Maj Institute of Pharmacology Polish Academy of Sciences, 12 Smętna Street, 31-343 Kraków, Poland

**Keywords:** Feedback sensitivity, Animal model, Alcohol, Rat, Genes, MAO-A

## Abstract

**Background:**

The results of our previous studies demonstrated that low sensitivity to negative feedback (NF) is associated with increased vulnerability to the development of compulsive alcohol-seeking in rats. In the present study, we investigated the molecular underpinnings of this relationship.

**Methods:**

Using TaqMan Gene Expression Array Cards, we analyzed the expression of the genes related to NF sensitivity and alcohol metabolism in three cortical regions (medial prefrontal cortex [mPFC], anterior cingulate cortex [ACC], orbitofrontal cortex [OFC]) and two subcortical regions (nucleus accumbens [Nacc], amygdala [Amy]). Gene expression differences were confirmed at the protein level with Western blot.

**Results:**

Sensitivity to NF was characterized by differences in *Gad2*, *Drd2*, and *Slc6a4* expression in the ACC, *Maoa* in the mPFC, and *Gria1*, *Htr3a*, and *Maoa* in the OFC. Chronic alcohol consumption was associated with differences in the expression of *Comt* and *Maoa* in the ACC, *Comt*, *Adh1*, and *Htr2b* in the mPFC, *Adh1,* and *Slc6a4* in the Nacc, *Gad2,* and *Htr1a* in the OFC, and *Drd2* in the Amy. Interactions between the sensitivity to NF and alcohol consumption were observed in the expression of *Gabra1*, *Gabbr2*, *Grin2a*, *Grin2b*, and *Grm3* in the ACC, and *Grin2a* in the OFC. The observed differences were confirmed at the protein level for MAO-A in the mPFC, and ADH1 in the mPFC and Nacc.

**Conclusions:**

Our findings contribute to a better understanding of the molecular mechanisms underlying the relationship between trait sensitivity to NF and compulsive alcohol consumption.

**Supplementary Information:**

The online version contains supplementary material available at 10.1007/s43440-023-00563-4.

## Introduction

Alcohol use disorder (AUD) is a chronic psychiatric condition characterized by the progression from occasional, moderate drinking to compulsive alcohol abuse. AUD is a significant global health issue, predominantly affecting men, leading to a high number of deaths each year. The economic burden of alcohol abuse on a global scale is enormous [1, 2]. The intricate nature of this disorder and the inter-individual differences between people suffering from alcohol dependence implies that individual traits may play a role in determining susceptibility to the development of compulsive drinking and subsequent addiction. Previous studies have indicated that people with symptoms of alcohol dependence often exhibit reduced responsiveness to the adverse consequences of their actions, as well as a decreased capacity to utilize negative feedback (NF) for regulating and adapting current behavior [3].This hints at a potential deficit in their feedback processing [3, 4]. Increased sensitivity to NF manifests itself in inadequate responses to negative outcomes of one’s actions and deficits in adjusting behavior following failures or errors [5, 6]. However, until recently, it remained unclear whether this biased processing preceded the onset of alcohol dependence or was a consequence of it.

In a recent publication from our laboratory [7], we presented findings highlighting the significant influence of trait sensitivity to NF on the development and maintenance of an alcohol-dependent-like state in rats. Our research demonstrated that trait sensitivity to NF can modulate alcohol-seeking behavior in response to punishment or the absence of expected rewards. Specifically, we found that rats with lower sensitivity to NF exhibited a higher propensity for compulsive alcohol-seeking compared to their more sensitive conspecifics. While these results shed light on the role of NF sensitivity in the development of an alcohol-dependent-like state, they did not elucidate the molecular mechanisms that could account for the observed effects.

Ethanol (EtOH) is a small, water-soluble molecule that is easily distributed throughout the body, allowing it to affect tissues and organs. The molecular effects of EtOH on the brain are intricate and encompass a multitude of mechanisms and signaling pathways. To gain further insight into the previously reported relationship between trait sensitivity to NF and prolonged alcohol consumption [7], the present study aimed to analyze differences in the expression of various genes in five brain regions: three cortical (medial prefrontal cortex [mPFC], anterior cingulate [ACC], and orbitofrontal cortex [OFC]) and two subcortical areas (nucleus accumbens [Nacc] and amygdala [Amy]). All the above-mentioned brain regions have been previously demonstrated to be involved in mediating sensitivity to feedback [8–11]. The selected genes were potentially linked to the modulation of NF sensitivity and the effects of alcohol. By extensively reviewing existing literature and analyzing the consequences of various genetic and pharmacological interventions on feedback sensitivity, four groups of genes were identified for scrutiny.

1. The first group encompassed genes responsible for the functioning and regulation of the serotonin (5-HT) system, such as serotonin receptors (5-HT_1A_, 5-HT_2A_), serotonin transporter (SERT), and tryptophan hydroxylase [12–15].
2. The second group of genes was selected based on their involvement in dopaminergic neurotransmission, as dopamine (DA) is the secondary neurotransmitter crucially implicated in feedback-based learning [10, 16]. This group included genes like dopamine receptors (D_1_, D_2_, D_4_), dopamine transporter (DAT), tyrosine hydroxylase, monoamine oxidase (MAO) A and B, and catechol-*O*-methyltransferase (COMT).
3. Because changes in brain DA neurotransmission often result from secondary neuroadaptations in other neurotransmitter systems, such as glutamate [17] and γ-aminobutyric acid (GABA) [18], genes associated with these 2 neurotransmitter systems, e.g., the ionotropic glutamate receptors NMDA and AMPA, the metabotropic glutamate receptors mGLU_2_, mGLU_3_, and mGLU_5_, glutamate decarboxylase (GAD), and GABA_A_ and GABA_B_ receptors, constituted the third analyzed group.
4. Genes implicated in EtOH metabolism, including catalase and alcohol dehydrogenase [19], constituted the fourth group.
5. Additionally, ribosomal protein L32 (*Rpl32*) and peptidylprolyl isomerase A (*Ppia*) were employed as reference genes, as described previously [20].

## Materials and methods

**Fig. 1:**
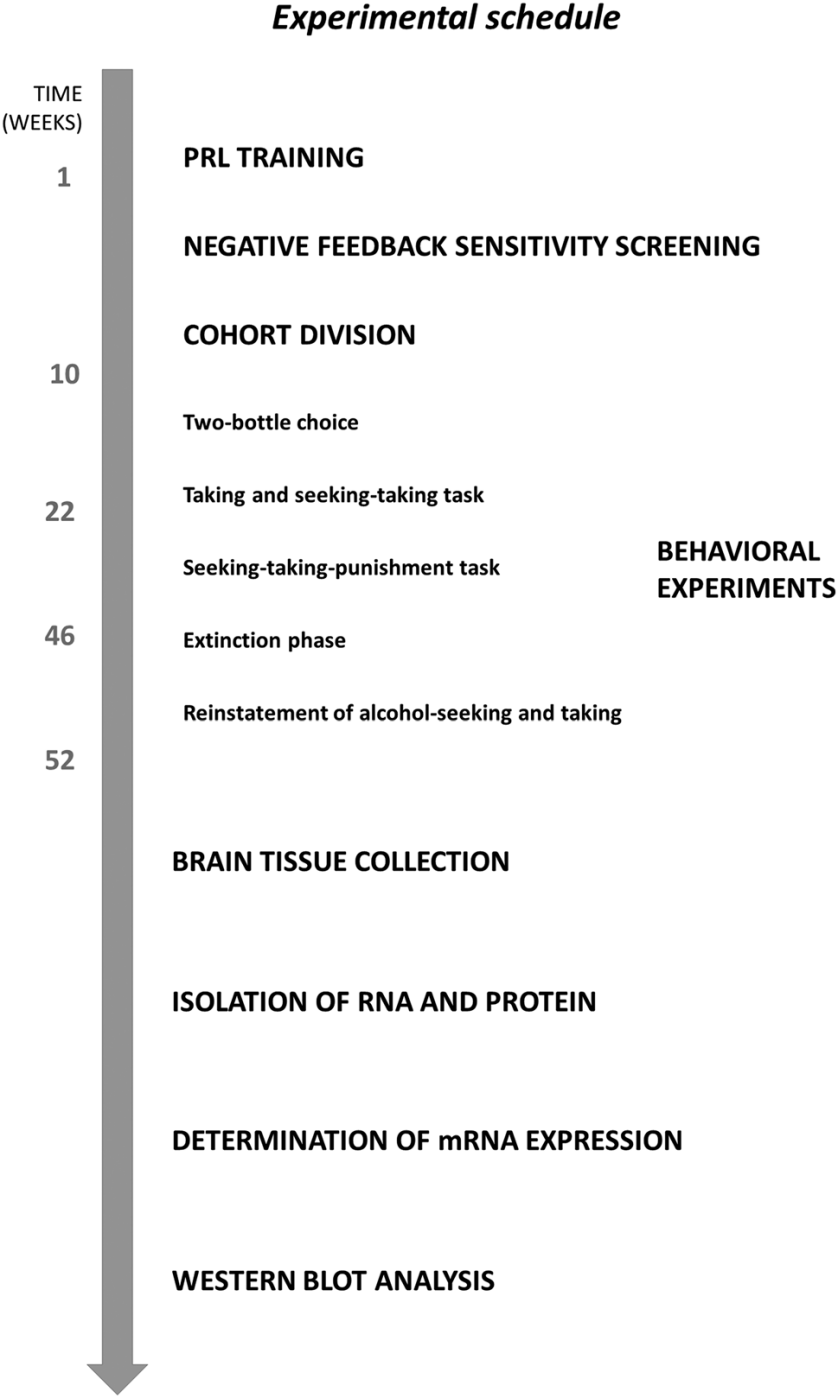
Experimental schedule. To determine the effects of lower and higher sensitivity to negative feedback (NF) and prolonged alcohol consumption on gene expression and protein levels, a cohort of rats was trained and tested in a series of Probabilistic Reversal Learning (PRL) tests. Based on this “Negative feedback sensitivity screening”, rats were classified as less sensitive and more sensitive to NF. The cohort was further divided into alcohol (EtOH) and water (H_2_O) drinking groups. Rats from the EtOH group were then subjected to a series of behavioral tests measuring hallmark symptoms of alcohol use disorder (behavioral data previously published [7]). H_2_O rats were handled daily throughout the entire experiment. At the end of these behavioral procedures, the rats were sacrificed, and the effects of prolonged alcohol consumption on gene expression and protein levels were compared between animals less sensitive and more sensitive to NF

In a previously published behavioral study, we analyzed differences in susceptibility to various aspects of compulsive alcohol consumption between 20 male Sprague–Dawley rats classified as less/more sensitive to NF [7]. This study had a non-drinking control group (*N* = 20) that could not be used for comparison in behavioral tests that used alcohol as a reward and was therefore not reported. In the current study, the brain tissue from these 20 additional rats along with the brain tissue of the 20 rats described in the previous study, was used to analyze the differences in the expression of a variety of genes related to feedback sensitivity and alcohol metabolism in rats with a lower and higher level of sensitivity to NF. This analysis was conducted within the groups of animals subjected to long-term exposure to alcohol and their non-drinking counterparts. The experimental schedule is summarized in Fig. 1.

### Ethical statement

All experiments were conducted following the European Union guidelines for the care and use of laboratory animals (2010/63/EU). Experimental protocols were reviewed and approved by the 2nd Local Institutional Animal Care and Use Committee, Institute of Pharmacology Polish Academy of Sciences in Krakow (Agreement: No. 230/2019, dated 10.10.2019). The authors declare that every effort has been made to minimize the animals’ suffering and the number of animals used.

### Subjects and behavioral procedures

We used 40 male Sprague–Dawley rats. Rats from the control group (*N* = 20) underwent probabilistic reversal learning (PRL) paradigm training together with EtOH rats (*N* = 20), for which the procedure was previously described in detail [7]. Briefly, the tests were conducted in the operant conditioning boxes, and each PRL session had 200 trials. During each trial, both levers were presented. One lever was randomly set as the “correct” lever, yielding an 80% reward rate, while the other, the “incorrect” lever, had a 20% reward rate. A 5 s intertrial interval (ITI) followed reward delivery. No response within 10 s was counted as an omission and also triggered the ITI. The same ITI followed unrewarded outcomes. After eight consecutive “correct” lever presses, the outcome probabilities were reversed. To evaluate rats' sensitivity to NF, indicating their ability to disregard occasional lack of reward, trial-by-trial decisions were monitored. Probabilistic lose-shifts, where rats switched levers after unrewarded “correct” lever press, were tallied as a ratio of all such outcomes on that lever. Using the results of 10 PRL tests from 10 consecutive days as a “sensitivity screening,” the rats were divided into two groups based on their sensitivity to NF, using the median to split them into less sensitive and more sensitive groups. This split was based on the average ratio of probabilistic in all 10 screening tests.

Rats from the EtOH group were then tested in a series of experiments measuring the hallmark features of alcohol addiction: alcohol intake, alcohol-seeking in the face of aversive consequences, and extinguishing and reinstating alcohol-seeking behavior. The results obtained during these behavioral procedures have been previously published along with a detailed description of the applied tests [7]. A detailed description of subjects, housing conditions, and behavioral procedures is presented in Supplementary materials S1. Control rats were handled daily for the entire duration of the experiment.

### Tissue collection

The day after the last alcohol intake and the last behavioral test, between 9:00 AM and 12:00 PM, the rats from both groups were decapitated in a counterbalanced manner (EtOH and control animal from the same NF sensitivity group at the same time), and five brain structures were collected for analyses: ACC, mPFC, Amy, Nacc, and OFC. Tissue was collected based on the “Rat Brain Atlas” of Paxinos and Watson [21] and according to Achterberg and colleagues [22]. The total number of samples for mRNA and protein analyses came from 34 animals: 18 less sensitive to NF (9 control and 9 EtOH) and 16 more sensitive to NF (10 control and 6 EtOH). The structures were frozen on dry ice and stored at −70 °C for further analysis.

### Isolation of RNA and protein from the brain structures

The purification procedure for total RNA isolated from collected tissues was performed according to the instructions provided with the commercially available RNeasy Plus Mini Kit (Qiagen, Germantown, MD, US). In addition, the protein was obtained during RNA isolation by cold acetone precipitation and then dissolved in urea buffer. The quality and quantity of the isolated total RNA were evaluated by a NanoDrop ND-1000 (Thermo Fisher Scientific) and an Experion microcapillary electrophoresis system (Bio-Rad, Hercules, California, US). Samples that passed the quality threshold (RIN > 8.0) were used for further experiments.

### Determination of mRNA expression by TaqMan Gene Expression Array Cards

The isolated RNAs were used to synthesize cDNA transcripts according to the manufacturer’s protocol of the High-Capacity cDNA Reverse Transcription Kit (Thermo Fisher Scientific). The amount of RNA was equalized for all samples depending on the structure. The obtained cDNA was mixed with TaqMan Universal PCR Master Mix, No AmpErase UNG (Thermo Fisher Scientific) for RT-qPCRs using Custom TaqMan Gene Expression Array Cards (Thermo Fisher Scientific). One Array Card was used to examine the mRNA expression of four samples in triplicate. The RT-qPCRs were run on a QuantStudio 12K Flex System (Applied Biosystems, Waltham, Massachusetts, US). Data were further analyzed with QuantStudio 12K Flex Software (Applied Biosystems). A Ct value above 34 was considered undetectable. The same threshold equal to 0.20 was set for all samples for comparison. Then, the data were analysed with qBasePLUS 3.1 software (Biogazelle, Zwijnaarde, Belgium) [23]. *Rpl32* and *Ppia* were selected for normalization.

### Western blot analysis

The concentration of proteins was determined using the Bradford Reagent (Sigma-Aldrich, Saint Louis, MO, USA) following the manufacturer’s protocol. Equal concentrations of proteins were mixed with 4 × Bolt® LDS Sample Buffer (Invitrogen, Waltham, MA, USA) and 10× Bolt® Sample Reducing Agent (Invitrogen) and then denatured at 70 °C for 10 min. Samples were separated on Bolt™ 4–12% Bis–Tris Plus Gels (Invitrogen) under reducing conditions in 20× Bolt® MES SDS Running Buffer (Invitrogen), incubated in 20% ethanol for 10 min, and transferred to immunoblot nitrocellulose membranes (iBlot® 2 Transfer Stacks, nitrocellulose, Invitrogen, Waltham, MA, USA) following the manufacturer’s protocol. Primary and secondary antibodies were suspended in an iBind™ Solution Kit followed by membrane incubation on iBind™ Cards using the iBind™ Western Device (SLF1000, Invitrogen, Waltham, MA, USA) for 2.5 h or overnight. Due to the lack of high-quality primary antibodies, we were unable to verify differences in the expression of several genes at the protein level. Western blot analysis was performed for the following proteins: MAO-A, ADH1, 5-HT_3A,_ and SERT. The following concentrations of primary antibodies were used to determine protein levels: 1:2000 for MAO-A (rabbit, cat. number: PA579623, Invitrogen)), 1:2000 for ADH1 (rabbit, cat. number PA5-78,730, Invitrogen), 1:1000 for 5-HT_3A_ (rabbit, cat. number: bs-2126R Bioss antibodies), and 1:2000 for SERT (rabbit, cat. number: PA5-80032, Invitrogen). The secondary anti-rabbit (cat. number: ab6721, Abcam) antibodies were used at concentrations of 1:20 000. As a loading control, β-actin (monoclonal anti-β-actin antibody produced in mouse, A5441, Sigma-Aldrich, Saint Louis, MO, USA) was applied at a concentration of 1:20 000, and its corresponding secondary antibody (anti-mouse IgG, A9044, Sigma‒Aldrich, Saint Louis, MO, USA) was applied at a concentration of 1:20 000. The electrophoretic bands were detected using the Clarity™ Western ECL Substrate (Bio-Rad, Hercules, CA, USA) and FUJIFILM LAS-4000 (Fujifilm Life Science, USA) device. Blot analysis was performed using ImageJ 1.53e software (Wayne Rusband and NIH, USA). Due to limited gel spots, a minimum of three samples from different groups were included in each blot.

### Statistics

The data were analyzed using SPSS (version 25.0, SPSS Inc., Chicago, IL, USA). The normality of the data was assessed using the Shapiro–Wilk test. For gene expression and protein level data, two-way ANOVAs were conducted. For pairwise comparisons, the values were compared using Sidak’s post-hoc tests. Nonparametric data were normalized by applying the square root transformation and, if necessary, outliers were removed. In cases where data could not be normalized, the Kruskal–Wallis test was employed followed by Dunn’s post hoc test. Feedback sensitivity screening data were analyzed using two-way repeated-measures ANOVA, with the within-subject factor being the test day/session and the between-subject factor being the sensitivity to NF. All significance tests were conducted with *α* = 0.05. The homogeneity of variance was examined using Levene's test, and for repeated-measures analyses, sphericity was confirmed using Mauchly's test. The data are presented as the mean ± SEM (standard error of the mean) for parametric data, or as the median and interquartile range for nonparametric data.

## Results

### NF sensitivity screening

**Fig. 2:**
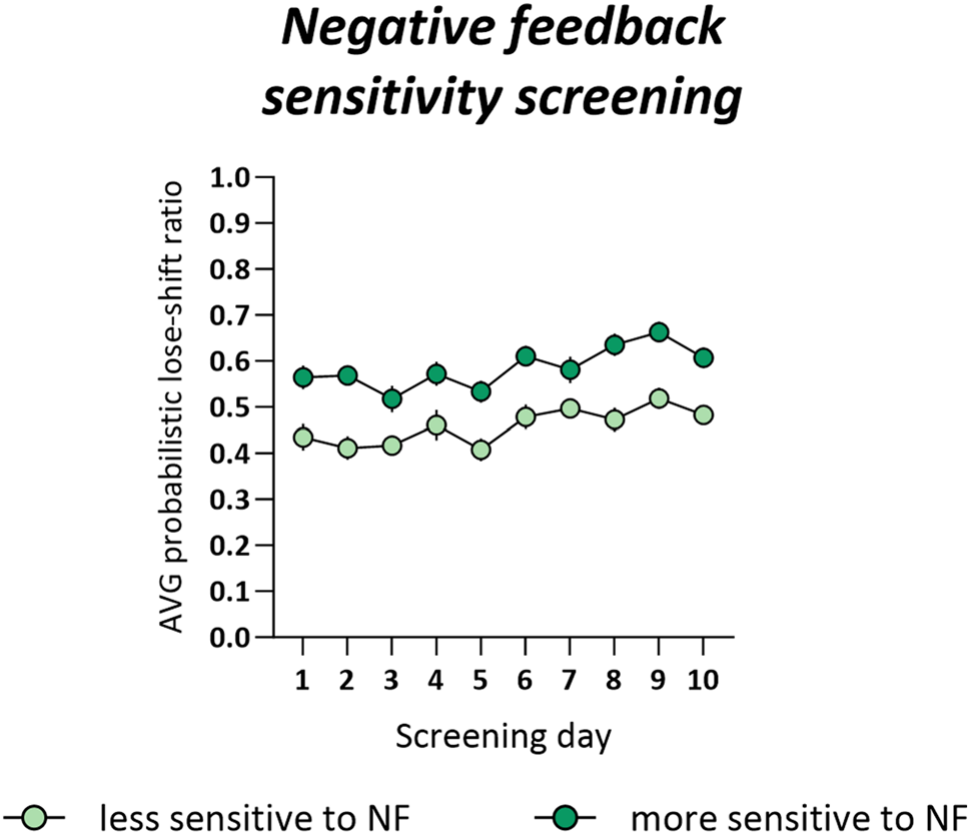
Negative feedback (NF) sensitivity screening. The average proportion of lose-shift behaviors following misleading unrewarded outcomes in rats classified as less sensitive (*n* = 20) and more sensitive to NF (*n* = 20) across all 10-screening probabilistic reversal learning tests. Data are presented as the mean ± SEM

All animals fulfilled the PRL training criteria and qualified for the PRL screening. Screening data for the EtOH group have been previously published [7]. Screening data for the whole cohort are presented in Fig. 2. For the animals classified as less sensitive to NF, the average proportion of lose-shift behaviors following misleading NF ranged from 0.36 to 0.54, with an average of 0.46 ± 0.01. For those classified as more sensitive to NF, the average proportion of probabilistic lose-shift behaviors ranged from 0.54 to 0.71, with an average of 0.59 ± 0.01. The difference in sensitivity to NF between both groups was stable across the screening period (non-significant interaction between screening day and NF sensitivity (*F*_9,342_ = 0.331, *p* = 0.542), a significant sensitivity effect (*F*_1,38_ = 62.36, *p* < 0.001), Fig. 2).

### Gene expression

**Fig. 3:**
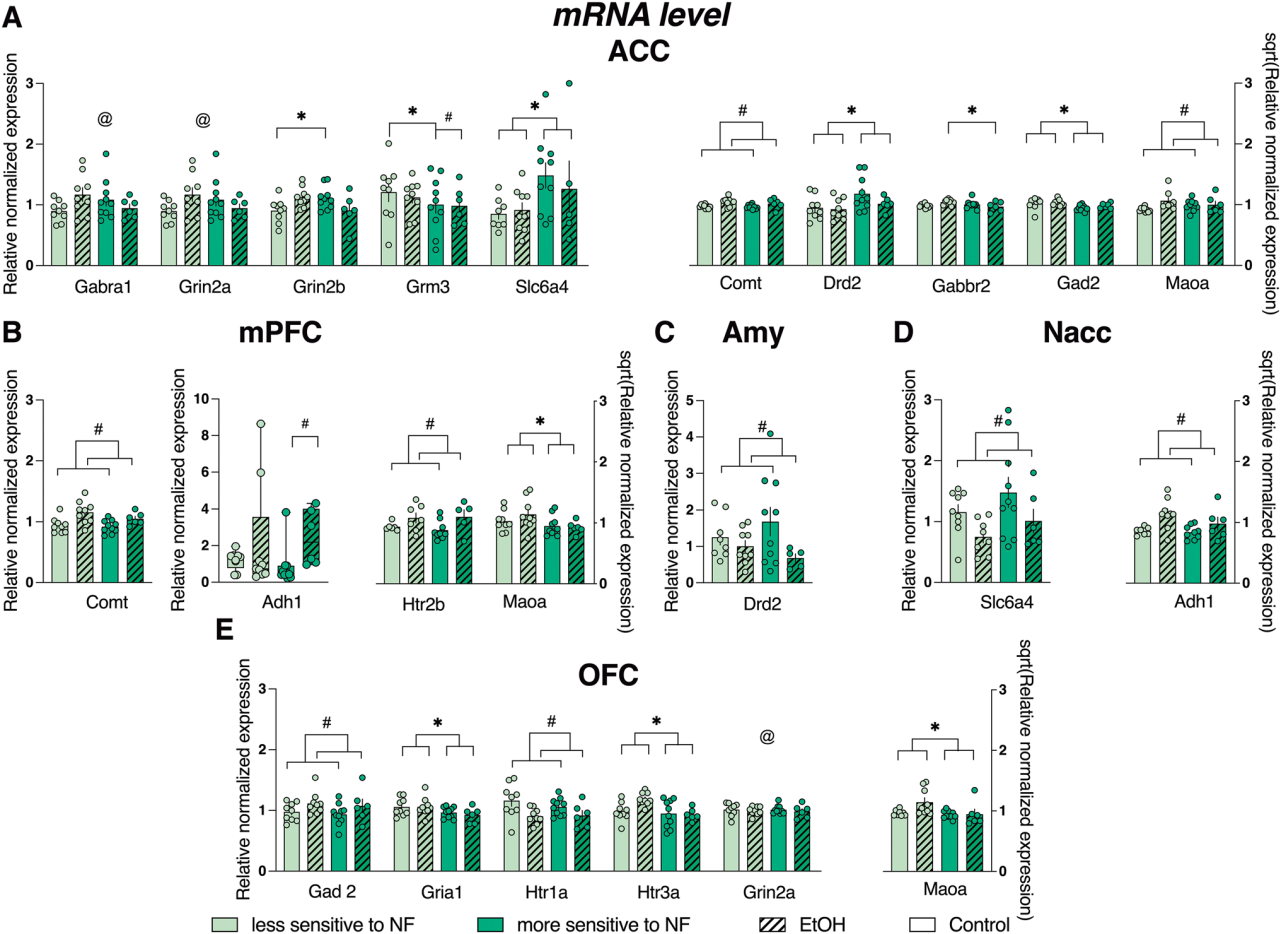
Genes expression following chronic alcohol exposure in the brains of male Sprague Dawley rats with higher or lower sensitivity to negative feedback (NF). Bar graphs represent a relative normalized expression of the genes assessed with TaqMan Gene Expression Array Cards in animals less sensitive to NF (light green bars) and more sensitive to NF (dark green bars) belonging to control (open bars) and EtOH (dashed bars) groups in **A** anterior cingulate cortex (ACC), **B** medial prefrontal cortex (mPFC), **C** amygdala (Amy), **D** nucleus accumbens (Nacc) and **E** orbitofrontal cortex (OFC); Total number of samples: *n* = 34 (less sensitive to NF: 9 control (8 in Amy), 9 EtOH; more sensitive to NF: 10 control, 6 EtOH [5 in Amy]). For some genes, single samples were excluded due to abnormalities in the gene expression readings or the removal of outliers during data normalization. The scale for normally distributed data is shown on the left *Y*-axis. The scale of the right *Y*-axis corresponds to the data normalized by square root transformation. Data are presented as the mean ± SEM (**A**–**E**) or as a median and interquartile range (B: *Adh1*) * indicates a significant (*p* < 0.05) difference between animals less and more sensitive to NF. # indicates a significant (*p* < 0.05) difference between the EtOH and control group. @ indicates significant NF sensitivity × alcohol exposure interaction with non-significant inter-group differences in post hoc tests (2-way ANOVA, Sidak’s post hoc test; for *Adh1* in mPFC Kruskal–Wallis test, Dunn’s post hoc test)

Analysis of the gene expression revealed statistically significant lower levels of mRNA in rats more sensitive to NF compared to the less sensitive group, for *Gad2* (F_1, 29_ = 7.533, *p* = 0.010) in ACC (Fig. 3A), for *Maoa* (F_1, 30_ = 5.229, *p* = 0.029) in mPFC (Fig. 3B) and *Gria1* (F_1, 30_ = 6.268, *p* = 0.018), *Htr3a* (*F*_1, 30_ = 6.514, *p* = 0.016), and *Maoa* (*F*_1, 29_ = 4.734, *p* = 0.038) in OFC (Fig. 3E).

In the ACC, the level of mRNA for *Drd2* (*F*_1, 30_ = 4.920, *p* = 0.034) and *Slc6a4* (*F*_1, 28_ = 5.254, *p* = 0.030) was significantly higher in the more sensitive to NF group (Fig. 3A). There were no significant effects of NF sensitivity on the expression of genes of interest in Amy and Nacc.

The mRNA levels were higher in the EtOH group compared to control, for *Comt* (*F*_1, 29_ = 10.220, *p* = 0.003) and *Maoa* (*F*_1, 29_ = 4.368, *p* = 0.046) in ACC, for *Comt* (*F*_1, 30_ = 13.270, *p* = 0.001), and *Htr2b* (*F*_1, 23_ = 6.437, *p* = 0.018) in mPFC (Fig. 3B), for *Adh1* (*F*_1, 27_ = 9.895, *p* = 0.004) in Nacc (Fig. 3D), and *Gad2* (F_1, 30_ = 4.390, *p* = 0.045) in OFC (Fig. 3E).

In mPFC, the mRNA level of *Adh1* was higher in the EtOH group than in the control group only for rats more sensitive to NF (Kruskal–Wallis test: *p* = 0.033, Fig. 3B). The expression of *Drd2* (*F*_1, 28_ = 4.436, *p* = 0.044) in Amy (Fig. 3C), *Slc6a4* (*F*_1, 29_ = 5.258, *p* = 0.029) in Nacc (Fig. 3D), and *Htr1a* (*F*_1, 30_ = 8.506, *p* = 0.007) in OFC (Fig. 3E) was lower in the EtOH group compared to the controls.

The analysis also revealed significant interactions between the effects of sensitivity to NF and the effects of prolonged alcohol exposure on the expression of *Gabra1* (*F*_1, 30_ = 4.629 *p* = 0.040), *Gabbr2* (*F*_1, 30_ = 5.772 *p* = 0.023), *Grin2a* (*F*_1, 30_ = 4.629, *p* = 0.040), *Grin2b* (*F*_1, 30_ = 9.156, *p* = 0.005), and *Grm3* (*F*_1, 30_ = 9.867, *p* = 0.004) in ACC (Fig. 3A), and on the expression of *Grin2a* (*F*_1, 30_ = 4.629, *p* = 0.040) in OFC (Fig. 3E). In the group of rats more sensitive to NF, the mRNA level of *Grm3* in ACC was lower in the EtOH group than in their control conspecifics. Additionally, within the control group, rats more sensitive to NF exhibited lower levels of *Grm3* expression compared to their less sensitive to NF counterparts. For *Grin2b* in ACC within the control group, rats more sensitive to NF showed higher mRNA expression compared to their less sensitive counterparts. For the EtOH group in ACC, the mRNA level of *Gabbr2* was lower in rats more sensitive to NF compared to their less sensitive conspecifics. The post-hoc tests did not reveal significant inter-group differences in the expression of *Garba1*, *Grin2a* in ACC, and *Grin2a* in OFC.

The results of statistical analyses of the expression of all genes are listed in Table S1 (Supplementary Materials S2). Two samples from Amy failed to pass the RNA quality threshold. Abnormalities in the gene expression readings were detected in certain samples on the RT-PCR card, specifically: for *Htr2b* in Amy, mPFC, ACC, NaCC, and OFC; for *Slc6a3* in Amy, mPFC, ACC, and OFC; and *Slc6a4* in ACC and Nacc. These results were not included in the analysis.

### Protein expression

**Fig. 4:**
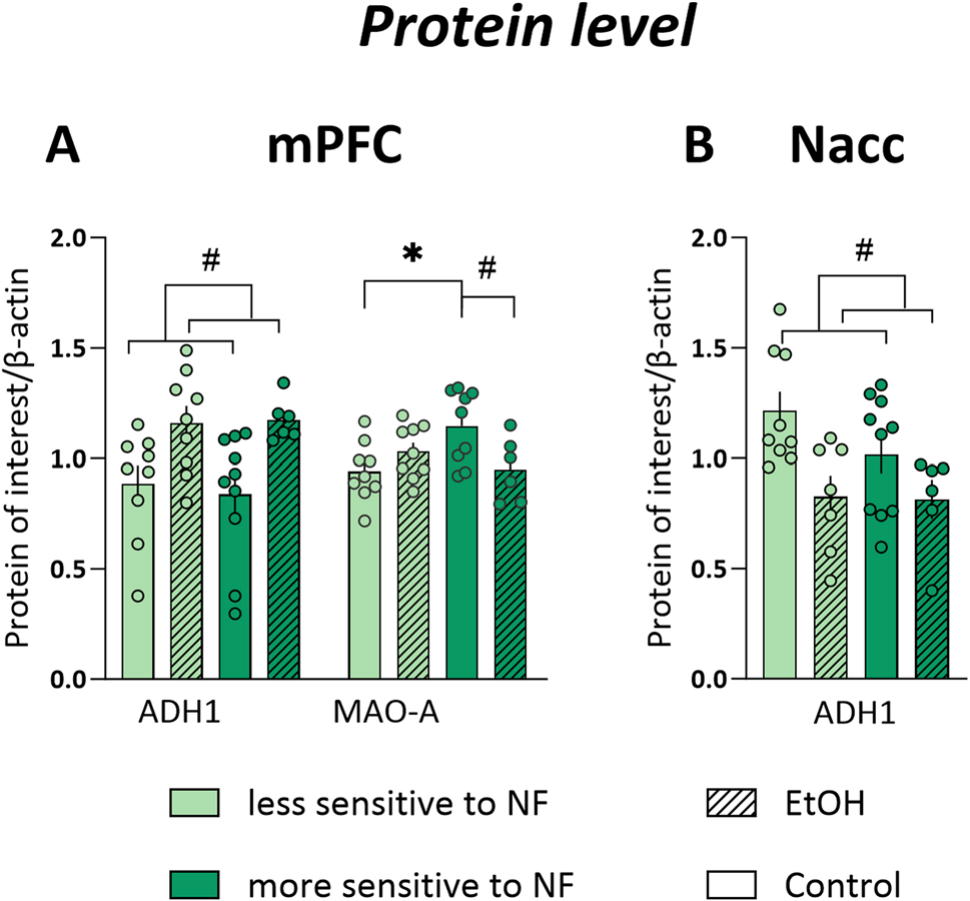
Protein levels following chronic alcohol exposure in the brains of male Sprague Dawley rats with higher or lower sensitivity to negative feedback (NF). **A** Alcohol dehydrogenase 1 (ADH1) and Monoamine oxidase A (MAO-A) to β-actin ratio in animals less sensitive to NF (light green bars) and more sensitive to NF (dark green bars) belonging to control (open bars) and EtOH (dashed bars) groups in mPFC **B**) ADH1 to β-actin ratio in animals less sensitive to NF (light green bars) and more sensitive to NF (dark green bars) belonging to control (open bars) and EtOH (dashed bars) groups in Nacc. Total number of samples included in Western blot analysis was n = 34 (less sensitive to NF: 9 control, 9 EtOH; more sensitive to NF: 10 control, 6 EtOH). For MAOA-A in mPFC, one sample was excluded (control, more sensitive to NF) and for ADH1 in Nacc, two samples (EtOH, less sensitive to NF) were excluded due to bands not being quantifiable. *Data are presented as the mean* ± *SEM.* * indicates a significant (*p* < 0.05) difference between animals less and more sensitive to NF # indicates a significant (*p* < 0.05) difference between the EtOH and control group (2-way ANOVA, Sidak’s post hoc test)

The observed differences in the mRNA levels were further explored at the protein level using the Western blot technique. Statistically significant effect of alcohol treatment on ADH1 protein (Alcohol dehydrogenase 1, gene: *Adh1*) level was detected in the mPFC (*F*_1,31_ = 7.650, *p* = 0.010; Fig. 4A) and in the Nacc (*F*_1,31_ = 7.650, *p* = 0.010; Fig. 4B).

Statistical analysis revealed also a significant interaction between the effects of sensitivity to NF and prolonged alcohol exposure on MAO-A protein level (Monoamine oxidase A, gene: *Maoa*) in mPFC (*F*_1,31_ = 7.650, *p* = 0.010; Fig. 4A), with a higher level of MAO-A in rats more sensitive to NF within the control group and significantly lower level of this protein in alcohol drinking group within the group of animals more sensitive to NF.

There were no statistically significant differences in the expression of other analyzed proteins. The results of statistical analyses of differences in the expression of all proteins of interest are listed in the Supplementary Table S2 (Supplementary materials S2). Original Western blot images are included in Supplementary materials S3. Some protein bands were unsuitable for quantification due to technical errors and are indicated by black frames (Supplementary materials S3).

## Discussion

The results of the study described above are complementary to and need to be discussed in the light of the results published in our earlier paper [7] in which we tested the hypothesis that in rats, individual vulnerability to compulsive seeking of alcohol may be linked to cognitive mechanisms based on sensitivity to NF. The experiments described in the mentioned study have confirmed the above assumption. Although initially the rats classified as NF less and more sensitive did not differ in voluntary alcohol consumption, the NF less sensitive animals turned out to be more vulnerable to compulsive alcohol seeking than their more NF-sensitive counterparts. This increased vulnerability was demonstrated by their weaker reaction to the unpredictable punishment of seeking responses (i.e., foot shock intensity increasing from 0.1 to 0.5 mA over repeated sessions) and their prolonged extinction of instrumental alcohol-seeking responses when alcohol was no longer available (for details see [7]).

The findings of our present study suggest that differences in the expression of certain genes and proteins, within several brain regions, can be linked to individual differences in sensitivity to NF, and the mechanisms determining the NF-linked vulnerability to compulsive alcohol-seeking and taking in rats. Specifically, we found that in the ACC, the rats that were more sensitive to NF had a lower level of mRNA expression of the *Gad2, a* gene, that is involved in the production of GABA [24], than their less NF-sensitive conspecifics. Conversely, rats that were more sensitive to NF had a higher mRNA expression level of *Drd2*, which encodes for a dopamine D_2_ receptor, and *Slc6a4*, which encodes for a serotonin transporter, than the rats from the NF less sensitive group. Similarly, in the mPFC and the OFC, the rats that were more sensitive to NF had a lower *Maoa* mRNA expression level than those, that were less sensitive to NF. Moreover, in the OFC, the level of mRNA expression of *Gria1*, which encodes for a subunit of a glutamate NMDA receptor, and the level of *Htr3a*, which encodes for a serotonin 5-HT_3A_ receptor, was lower in rats more sensitive to NF than in their NF less sensitive counterparts.

Our study also confirmed that chronic alcohol exposure leads to significant changes in gene expression in different brain regions of rats, which may contribute to the behavioral and physiological effects of alcohol. In the ACC, rats exposed to prolonged alcohol consumption had significantly higher mRNA levels of *Comt* and *Maoa* as compared to their non-drinking controls. *Comt* encodes for catechol *O*-methyl-transferase (COMT), an enzyme involved in the breakdown of DA and other catecholamines [25], while *Maoa* encodes for monoamine oxidase A (MAO-A), an enzyme involved in the breakdown of neurotransmitters such as 5-HT and DA [26]. Higher levels of these enzymes may reflect the increased activity of the neurotransmitter systems they break down, possibly as a compensatory response to chronic alcohol exposure. In the mPFC, the mRNA levels of *Comt* and *Htr2b*, which encodes for a serotonin 5-HT_2B_ receptor, were higher in the rats from the EtOH group as compared to their non-drinking controls. In the Nacc, alcohol-exposed rats had significantly higher levels of *Adh1*, which encodes for an alcohol dehydrogenase enzyme, and lower levels of the mentioned already above *Slc6a4*. In the OFC, alcohol-exposed rats had higher mRNA levels for *Gad2*, compared to control rats. However, they also had lower levels of *Htr1a*, which encodes for the serotonin 5-HT_1A_ receptor.

Our findings also showed that there are significant interactions between the sensitivity to NF and the effects of prolonged alcohol exposure on the expression of specific genes in different cortical regions, namely the ACC and OFC. The expression levels of *Gabra1, Gabbr2, Grin2a, Grin2b*, and *Grm3* were found to be significantly affected by both sensitivity to NF and prolonged alcohol exposure in the ACC. *Gabra1* and *Gabbr2* are involved in the regulation of GABA, while *Grin2a*, *Grin2b*, and *Grm3* are involved in glutamatergic neurotransmission. In the OFC, only the expression level of *Grin2a* was found to be affected by both sensitivity to NF and prolonged alcohol exposure. Alcohol has the potential to disrupt the delicate balance between GABA, the major inhibitory neurotransmitter, and glutamate, the principal excitatory neurotransmitter within the central nervous system [27]. The differences in gene expression related to GABAergic neurotransmission observed in ACC align with prior research findings that have demonstrated how alterations in GABA signaling can influence reward processes and the reinforcing effects of alcohol. Given that the mRNA expression analysis was conducted after a period of forced abstinence and reinstatement in the EtOH group, it should come as no surprise that there are differences in the expression of genes related to the glutamatergic system. In fact, numerous studies have indicated that alcohol withdrawal is associated with disturbances in excitatory amino acid transmission, and modulating it can alleviate withdrawal symptoms [28, 29].

In our investigation, most inter-trait differences in gene expression failed to manifest at the protein level. The lack of alignment between differences in gene expression and protein levels is not entirely surprising and could have several reasons, including post-transcriptional modifications, alternative splicing, translational regulation, and post-translational modifications. These processes introduce complexities that can obscure the direct relationship between gene activity and protein abundance and require further investigation. Despite this, we confirmed that animals more sensitive to NF within the control group had higher levels of MAO-A in mPFC than their NF less sensitive conspecifics. Though the precise function of MAO-A in influencing sensitivity to feedback remains uncertain, its heightened activity could potentially accelerate the breakdown of biogenic amines. This, in turn, may reduce their accessibility to receptors and hinder the processing of adverse information. It is worth mentioning that reversible monoamine oxidase inhibitors are commonly used in the treatment of depression and may potentially reduce sensitivity to NF, a trait commonly observed in individuals with depression [30–32]. Additionally, our study found that in animals more sensitive to NF, chronic alcohol consumption led to lower levels of MAO-A in the mPFC. This suggests that alcohol consumption downregulates MAO-A expression only in animals with higher sensitivity to NF, not in those with lower NF sensitivity. These findings are in line with previously published behavioral data where rats more sensitive to NF were less likely to seek alcohol when it was associated with punishment and after the termination of alcohol availability, compared to their less sensitive conspecifics [7]. Previous studies have shown that genetic variants of *Maoa* and epigenetic mechanisms are strongly associated with the occurrence of AUD in both humans and animals [33–35]. Although the exact mechanism by which alcohol regulates MAO-A expression is unknown, this finding presents a promising avenue for further research in identifying individual differences between animals less and more sensitive to NF and their susceptibility to the development of alcohol dependence. Future studies should aim to investigate the cellular mechanisms underlying MAO-A-driven susceptibility to alcohol dependence and explore epigenetic and regulatory mechanisms that may mediate the effects of chronic alcohol exposure on *Maoa* expression.

The second gene, the differences in expression of which were confirmed at the protein level, was the gene encoding alcohol dehydrogenase. Although alcohol-induced differences in the expression of this gene and protein levels were not unexpected, their presence confirms the effectiveness of the model used and positively verifies the effects of alcohol.

In conclusion, this study provides further evidence for the relationship between trait sensitivity to NF and compulsive alcohol consumption in rats. Our findings demonstrate significant differences in the expression of genes and (some) proteins related to NF sensitivity and alcohol metabolism in various cortical and subcortical regions of the brain between rats less and more sensitive to NF that consumed alcohol and their non-drinking counterparts. Because of the wide range of neurotransmitter and neuromodulator systems affected by alcohol, the effectiveness of current pharmacotherapies aimed at treating alcohol dependence is constrained. The imperative for reducing the harmful use of alcohol in a public health context requires the development of successful therapeutic strategies. Our research aimed to address this need by identifying potential molecular targets for new drugs to treat AUD. Our findings contribute to a better understanding of the molecular mechanisms underlying compulsive alcohol consumption in rats and therefore may have implications for the development of treatments for alcohol use disorders.

### Supplementary Information

Below is the link to the electronic supplementary material.

Supplementary file1 (DOCX 30 KB)

Supplementary file2 (DOCX 48 KB)

Supplementary file3 (DOCX 10555 KB)

## Data Availability

The data that support the findings of this study are available from the corresponding author, [RR], upon reasonable request.

## References

[1] Rehm J, Imtiaz S (2016). A narrative review of alcohol consumption as a risk factor for global burden of disease. Subst Abuse Treat Prev Policy.

[2] Molina PE, Gardner JD, Souza-Smith FM, Whitaker AM (2014). Alcohol abuse: critical pathophysiological processes and contribution to disease burden. Physiology (Bethesda).

[3] Bechara A, Damasio H (2002). Decision-making and addiction (part I): impaired activation of somatic states in substance dependent individuals when pondering decisions with negative future consequences. Neuropsychologia.

[4] Kamarajan C, Rangaswamy M, Tang Y, Chorlian DB, Pandey AK, Roopesh BN (2010). Dysfunctional reward processing in male alcoholics: an ERP study during a gambling task. J Psychiatry Res.

[5] Santesso DL, Steele KT, Bogdan R, Holmes AJ, Deveney CM, Meites TM (2008). Enhanced negative feedback responses in remitted depression. NeuroReport.

[6] Ruchsow M, Herrnberger B, Beschoner P, Grön G, Spitzer M, Kiefer M (2006). Error processing in major depressive disorder: evidence from event-related potentials. J Psychiatry Res.

[7] Cieslik A, Noworyta K, Rygula R (2022). Trait sensitivity to negative feedback determines the intensity of compulsive alcohol seeking and taking in male rats. J Psychiatry Neurosci.

[8] Clarke HF, Cardinal RN, Rygula R, Hong YT, Fryer TD, Sawiak SJ (2014). Orbitofrontal dopamine depletion upregulates caudate dopamine and alters behavior via changes in reinforcement sensitivity. J Neurosci.

[9] Cools R, Clark L, Owen AM, Robbins TW (2002). Defining the neural mechanisms of probabilistic reversal learning using event-related functional magnetic resonance imaging. J Neurosci.

[10] Cools R, Frank MJ, Gibbs SE, Miyakawa A, Jagust W, D'Esposito M (2009). Striatal dopamine predicts outcome-specific reversal learning and its sensitivity to dopaminergic drug administration. J Neurosci.

[11] Dalton GL, Phillips AG, Floresco SB (2014). Preferential involvement by nucleus accumbens shell in mediating probabilistic learning and reversal shifts. J Neurosci.

[12] Chamberlain SR, Müller U, Blackwell AD, Clark L, Robbins TW, Sahakian BJ (2006). Neurochemical modulation of response inhibition and probabilistic learning in humans. Science.

[13] Cools R, Roberts AC, Robbins TW (2008). Serotoninergic regulation of emotional and behavioural control processes. Trends Cogn Sci.

[14] Rygula R, Clarke HF, Cardinal RN, Cockcroft GJ, Xia J, Dalley JW (2015). Role of central serotonin in anticipation of rewarding and punishing outcomes: effects of selective amygdala or orbitofrontal 5-HT depletion. Cereb Cortex.

[15] Golebiowska J, Rygula R (2017). Effects of acute dopaminergic and serotonergic manipulations in the ACI paradigm depend on the basal valence of cognitive judgement bias in rats. Behav Brain Res.

[16] Pessiglione M, Seymour B, Flandin G, Dolan RJ, Frith CD (2006). Dopamine-dependent prediction errors underpin reward-seeking behaviour in humans. Nature.

[17] Kauer JA, Malenka RC (2007). Synaptic plasticity and addiction. Nat Rev Neurosci.

[18] Volkow ND, Wang GJ, Fowler JS, Tomasi D, Telang F, Baler R (2010). Addiction: decreased reward sensitivity and increased expectation sensitivity conspire to overwhelm the brain's control circuit. BioEssays.

[19] Hipólito L, Sánchez MJ, Polache A, Granero L (2007). Brain metabolism of ethanol and alcoholism: an update. Curr Drug Metab.

[20] Gąska M, Kuśmider M, Solich J, Faron-Górecka A, Krawczyk MJ, Kułakowski K (2012). Analysis of region-specific changes in gene expression upon treatment with citalopram and desipramine reveals temporal dynamics in response to antidepressant drugs at the transcriptome level. Psychopharmacology.

[21] Paxinos G, Watson C (2006). The rat brain in stereotaxic coordinates: hard.

[22] Achterberg EJ, van Kerkhof LW, Damsteegt R, Trezza V, Vanderschuren LJ (2015). Methylphenidate and atomoxetine inhibit social play behavior through prefrontal and subcortical limbic mechanisms in rats. J Neurosci.

[23] Hellemans J, Mortier G, De Paepe A, Speleman F, Vandesompele J (2007). qBase relative quantification framework and software for management and automated analysis of real-time quantitative PCR data. Genome Biol.

[24] Erlander MG, Tillakaratne NJ, Feldblum S, Patel N, Tobin AJ (1991). Two genes encode distinct glutamate decarboxylases. Neuron.

[25] Männistö PT, Ulmanen I, Lundström K, Taskinen J, Tenhunen J, Tilgmann C (1992). Characteristics of catechol O-methyl-transferase (COMT) and properties of selective COMT inhibitors. Prog Drug Res.

[26] Abell CW, Kwan SW (2001). Molecular characterization of monoamine oxidases A and B. Prog Nucleic Acid Res Mol Biol.

[27] Valenzuela CF (1997). Alcohol and neurotransmitter interactions. Alcohol Health Res World.

[28] Narita M, Soma M, Mizoguchi H, Tseng LF, Suzuki T (2000). Implications of the NR2B subunit-containing NMDA receptor localized in mouse limbic forebrain in ethanol dependence. Eur J Pharmacol.

[29] Bienkowski P, Krzascik P, Koros E, Kostowski W, Scinska A, Danysz W (2001). Effects of a novel uncompetitive NMDA receptor antagonist, MRZ 2/579 on ethanol self-administration and ethanol withdrawal seizures in the rat. Eur J Pharmacol.

[30] Beats BC, Sahakian BJ, Levy R (1996). Cognitive performance in tests sensitive to frontal lobe dysfunction in the elderly depressed. Psychol Med.

[31] Taylor Tavares JV, Drevets WC, Sahakian BJ (2003). Cognition in mania and depression. Psychol Med.

[32] Taylor Tavares JV, Clark L, Furey ML, Williams GB, Sahakian BJ, Drevets WC (2008). Neural basis of abnormal response to negative feedback in unmedicated mood disorders. Neuroimage.

[33] Philibert RA, Sandhu H, Hollenbeck N, Gunter T, Adams W, Madan A (2008). The relationship of 5HTT (SLC6A4) methylation and genotype on mRNA expression and liability to major depression and alcohol dependence in subjects from the Iowa Adoption Studies. Am J Med Genet B Neuropsychiatr Genet.

[34] Cervera-Juanes R, Wilhem LJ, Park B, Lee R, Locke J, Helms C (2016). MAOA expression predicts vulnerability for alcohol use. Mol Psychiatry.

[35] Bendre M, Granholm L, Drennan R, Meyer A, Yan L, Nilsson KW (2019). Early life stress and voluntary alcohol consumption in relation to Maoa methylation in male rats. Alcohol.

