# Supplementary material for: Identification of genes regulated by trait sensitivity to negative feedback and prolonged alcohol consumption in rats
Source: Pharmacol Rep. 2024 Jan 3;76(1):207–15. doi: 10.1007/s43440-023-00563-4 (PMC10830829; doi:10.1007/s43440-023-00563-4)
Supplement: Supplementary file 1 — Supplementary file1 (DOCX 30 KB) [file 43440_2023_563_MOESM1_ESM.docx]

# **Supplementary materials S1**

# **Methods**

## *Subjects and housing*

We used male Sprague Dawley rats housed in groups of 4 rats per cage, in an enriched environment (wooden blocks and plastic pipes 25 cm long), with controlled temperature (21 ± 1 °C) and humidity (40 - 50%) and water always available *ad libitum*. Both groups of rats (EtOH and control) were delivered on the same day with an initial body weight of 176–200 g (5 weeks old according to the Growth Chart provided by the laboratory rodent supplier Charles River). They were kept on a 12-hour light/dark cycle, with lights turned on at 7:00 AM. All procedures were conducted during the light phase. The rats were mildly food-restricted throughout the experiment to facilitate instrumental training [1-3], receiving 15 g of standard laboratory chow per day, which was equivalent to 85% of their free-feeding weight based on the normal growth curve recommended by the laboratory rodent supplier, Charles River Research Models and Services Catalogue.

## *Experimental Apparatus*

PRL tests were conducted in operant conditioning chambers (Med Associates; St Albans, Vermont, USA) enclosed within a sound-attenuating box. Each chamber was equipped with a fan, house light, speaker, a food dispenser set to deliver a sucrose pellet (Dustless Precision Pellets, 45 mg; Bio-Serv, New Jersey, USA), fluid receptacle, and two retractable levers located at the sides of the feeder.

## *Measuring sensitivity to feedback using the PRL test*

After the initial instrumental training described in detail elsewhere [4] and upon reaching the initial training criterion of less than 7.5% omissions on each lever (i.e., less than 15% total omissions but equally distributed between the 2 levers) for 3 consecutive training days, the rats from both groups were trained in the PRL paradigm. In brief, each PRL training session consisted of 200 trials, and each trial lasted for a maximum of 22 s. The start of a trial was signaled by the house light, which remained on until the end of the trial. Two seconds after the trial had started, both levers were presented, and one of them was randomly assigned as the “correct” lever, which delivered a reward (one sucrose pellet) 80% of the time it was pressed. A press on the other lever - the “incorrect” lever - would result in a rewarding outcome only 20% of the time it was pressed. A 5 s intertrial interval (ITI) followed reward delivery. During the ITI, both levers remained retracted, and the house light was turned off. No response in 10 s triggered the ITI and was counted as an omission. The same ITI directly followed an unrewarded outcome, i.e., no reward on 20% of the “correct” and 80% of the “incorrect” lever presses. After every 8 consecutive “correct” lever presses (regardless of the outcome), the criterion for the reversal of the outcome probabilities was reached. The previously “correct” lever now became “incorrect” and vice versa. This pattern was followed until the end of the session. The PRL training phase was repeated daily until the individual animals achieved sufficient performance levels. The criteria to be met were a minimum of 3 reversals completed during 3 consecutive training sessions, with less than 15% omissions per session.

## *Parameters measured in the PRL test*

To assess rats' sensitivity to negative feedback (NF), which reflects their ability to disregard occasional and misleading lack of reward, their decisions were tracked trial by trial. The number of unrewarded outcomes for the "correct" lever that were followed by the animal switching to the other lever (probabilistic lose-shifts) were recorded, and expressed as a ratio of all unrewarded outcomes for that lever. To measure rats' sensitivity to positive feedback, all rewarded outcomes (both true and misleading) that were followed by a decision to stick with the lever that produced them (win-stays) were counted for both the "correct" and "incorrect" levers, and expressed as a ratio of all rewarded outcomes for that lever. This method of analyzing sensitivity to positive feedback was inspired by Bari et al.'s approach and was based on the infrequency of win-stay behavior after misleading rewards on the incorrect lever[2](2)(2)(2)(2)(3). The number of reversals completed during the test was used as an indicator of the animals' performance.

*Feedback sensitivity screening*

Once the rats achieved a stable performance in the PRL test, with a minimum of 3 reversals and less than 15% omissions in three consecutive sessions, they underwent 10 consecutive PRL tests over 10 days. Using the results of these tests as a "sensitivity screening," the rats were divided into two groups based on their sensitivity to NF, using the median to split them into less sensitive and more sensitive groups. This division was determined by calculating the average ratio of lever changes following misleading unrewarded outcomes (probabilistic lose-shifts) made by the animals across all 10 screening tests.

*Procedures measuring alcohol-related behaviors*

### *Intermittent access 2BC paradigm*

To induce drinking behavior and to determine the level of alcohol consumption in the rats, 18 sessions of the intermittent access 2BC procedure were conducted every second day. During the 2BC test, animals were separated into individual cages for 24 hours, where they were presented with one bottle of 10% ethanol (EtOH) (w/w) and one bottle of water. The bottles were weighed before and after each session to determine alcohol consumption (g EtOH/kg).

### *TAKING TASK*

Initially, the rats were trained to associate the pressing of the taking lever with alcohol delivery under a fixed-ratio 1 (FR1) schedule of reinforcement. Each trial started with the insertion of the randomly assigned taking lever and the house light on. Pressing on the lever resulted in the dipper presentation on the opposite side of the box, delivery of 0.1 ml of 15% EtOH (w/w), and simultaneous retraction of the taking lever. Rats were limited to a maximum of 60 rewards for a 30 min training session. After achieving the performance criterion of a minimum of 20 taking responses in 3 consecutive sessions, the animals were shifted to the ST phase of the training.

### *ST TASK*

During this task, each trial started with the insertion of the seeking lever, next to the previously assigned taking lever, which remained retracted. Pressing on the seeking lever led to the extension of the taking lever following a random interval of 1 to 15 s (RI 1-15 s). Pressing on the taking lever resulted in the presentation of the dipper on the opposite side of the box, delivery of 0.1 ml of 15% EtOH (w/w), and simultaneous retraction of both levers. Rats were limited to a maximum of 100 rewards for a 45 min session. After achieving the performance criterion of a minimum of 20 taking responses in 3 consecutive sessions, the animals were ready to be tested on the seeking taking punishment (STP) task*.*

### *STP TASK*

In this paradigm, each trial started as described for the ST task, with the insertion of the seeking lever. The seeking lever response resulted either in a 1 s electric shock (0.10-0.50 mA), administered through a grid floor, or the extension of the taking lever after a random interval (RI 1-15 s). Each session consisted of 25 trials, of which 8 (30%) were punished with foot shock and 17 (70%) were reinforced by the delivery of 0.1 ml 15% EtOH following the taking lever response. The intensity of the shock increased gradually in the consecutive test sessions according to the following pattern: 0.10, 0.20, 0.30, 0.30, 0.40, 0.40, 0.50, and 0.50 mA. Although punishment occurred randomly in each session, never more than two consecutive trials resulted in a foot shock, and the first trial of the session was always reinforced.

### *Extinction of alcohol-seeking and taking behaviors*

After the completion of STP testing, the animals underwent 5 additional ST tests (baseline) and were then subjected to daily extinction sessions (lasting 15 min), during which the seeking lever response resulted in the extension of the taking lever (under RI 1-15 s); however, the pressing of that lever had no programmed consequences, and alcohol was not available. After reaching less than 5 seeking responses in 3 consecutive sessions, the rats were not tested for the following 30 days of alcohol abstinence.

### *Reinstatement of alcohol-seeking and taking behaviors*

After the 30 days of abstinence, the rats underwent a series of ST tests to measure how quickly they reinstated their alcohol-seeking behavior. The animals were tested until they reached the criterion of an average number of seeking responses from 5 tests that was equal to or higher than the average number of seeking responses from the 5 baseline ST tests.

1. Boulougouris, V., A. Castañé, and T.W. Robbins, *Dopamine D2/D3 receptor agonist quinpirole impairs spatial reversal learning in rats: investigation of D3 receptor involvement in persistent behavior.* Psychopharmacology (Berl), 2009. **202**(4): p. 611-20.

2. Bari, A., et al., *Serotonin modulates sensitivity to reward and negative feedback in a probabilistic reversal learning task in rats.* Neuropsychopharmacology, 2010. **35**(6): p. 1290-301.

3. Izquierdo, A., et al., *Genetic and dopaminergic modulation of reversal learning in a touchscreen-based operant procedure for mice.* Behav Brain Res, 2006. **171**(2): p. 181-8.

4. Noworyta-Sokolowska, K., et al., *Sensitivity to negative and positive feedback as a stable and enduring behavioural trait in rats.* Psychopharmacology, 2019. **236**(8): p. 2389-2403.
