# Supplementary material for: Identification of genes regulated by trait sensitivity to negative feedback and prolonged alcohol consumption in rats
Source: Pharmacol Rep. 2024 Jan 3;76(1):207–15. doi: 10.1007/s43440-023-00563-4 (PMC10830829; doi:10.1007/s43440-023-00563-4)
Supplement: Supplementary file 2 — Supplementary file2 (DOCX 48 KB) [file 43440_2023_563_MOESM2_ESM.docx]

**Table S1. The effects of trait sensitivity to NF and alcohol drinking on gene expression.**

Statistically significant effects and interactions are bolded (2-way ANOVA).

| **Structure** | **Gene name** | ***n*** | ***Interaction*** | ***Treatment*** | **Sensitivity** |
| --- | --- | --- | --- | --- | --- |
| **ACC** | *Adh1* | 31 | F _1, 27_ = 2.658  p = 0.115 | F _1, 27_ = 1.267  p = 0.253 | F _1, 27_ = 3.765  p = 0.063 |
|  | *Cat* | 34 | F _1, 30_ = 2.230  p = 0.137 | F _1, 30_ = 0.670  p = 0.419 | F _1, 30_ = 3.746  p = 0.062 |
|  | ***Comt*** | 33 | F _1, 29_ = 0.166  p = 0.686 | **F _1, 29_ = 10.22**  **p =** **0.003 **** | F _1, 29_ = 0.728  p = 0.401 |
|  | *Drd1* | 34 | F _1, 30_ = 1.759  p = 0.195 | F _1, 30_ = 0.235  p = 0.631 | F _1, 30_ = 1.128  p = 0.297 |
|  | ***Drd2*** | 34 | F _1, 30_ = 0.988  p = 0.328 | F _1, 30_ = 1.665  p = 0.207 | **F _1, 30_ = 4.920**  **p = 0.034 *** |
|  | *Gabbr1* | 34 | F _1, 30_ = 1.164  p = 0.289 | F _1, 30_ = 1.660  p = 0.207 | F _1, 30_ = 0.060  p = 0.808 |
|  | ***Gabbr2*** | 34 | **F _1, 30_ = 5.772**  **p = 0.023 *** | F _1, 30_ = 0.507  p = 0.482 | F _1, 30_ = 1.358  p = 0.253 |
|  | ***Gabra1*** | 34 | **F _1, 30_ = 4.629**  **p = 0.040 *** | F _1, 30_ = 0.492  p = 0.488 | F _1, 30_ = 0.036  p = 0.851 |
|  | *Gad1* | 34 | F _1, 30_ = 2.303  p = 0.140 | F _1, 30_ = 0.272  p = 0.606 | F _1, 30_ = 1.177  p = 0.287 |
|  | ***Gad2*** | 33 | F _1, 29_ = 0.317  p = 0.578 | F _1, 29_ = 1.259  p = 0.271 | **F _1, 29_ = 7.533**  **p = 0.01 *** |
|  | *Gria1* | 34 | F _1, 30_ = 0.883  p = 0.355 | F _1, 30_ = 1.352  p = 0.254 | F _1, 30_ = 2.289  p = 0.141 |
|  | ***Grin2a*** | 34 | **F _1, 30_ = 4.629**  **p = 0.04 *** | F _1, 30_ = 0.492  p = 0.488 | F _1, 30_ = 0.036  p = 0.851 |
|  | ***Grin2b*** | 34 | **F _1, 30_ = 9.156**  **p = 0.005 **** | F _1, 30_ = 0.038  p = 0.847 | F _1, 30_ = 0.011  p = 0.915 |
|  | *Grm2* | 34 | F _1, 30_ = 0.059  p = 0.810 | F _1, 30_ = 0.144  p = 0.707 | F _1, 30_ = 1.548  p = 0.223 |
|  | ***Grm3*** | 34 | **F _1, 30_ = 9.867**  **p = 0.004 **** | F _1, 30_ = 0.283  p = 0.599 | F _1, 30_ = 0.305  p = 0.585 |
|  | *Grm5* | 34 | F _1, 30_ = 0.013  p = 0.910 | F _1, 30_ = 0.480  p = 0.494 | F _1, 30_ = 0.564  p = 0.459 |
|  | *Htr1a* | 34 | F _1, 30_ = 1.159  p = 0.290 | F _1, 30_ = 3.805  p = 0.061 | F _1, 30_ = 0.020  p = 0.887 |
|  | *Htr2a* | 34 | F _1, 30_ = 0.268  p = 0.609 | F _1, 30_ = 0.046  p = 0.831 | F _1, 30_ = 0.360  p = 0.553 |
|  | *Htr2b* | 20 | F _1, 16_ = 0.574  p = 0.460 | F _1, 16_ = 0.045  p = 0.835 | F _1, 16_ = 0.187  p = 0.671 |
|  | *Htr3a* | 34 | F _1, 30_ = 1.068  p = 0.310 | F _1, 30_ = 0.042  p = 0.840 | F _1, 30_ = 0.335  p = 0.567 |
|  | *Npy* | 34 | F _1, 30_ = 2.918  p = 0.098 | F _1, 30_ = 1.631  p = 0.211 | F _1, 30_ = 0.482  p = 0.493 |
|  | ***Maoa*** | 33 | F _1, 29_ = 2.956  p = 0.096 | **F _1, 29_ = 4.368**  **p =** **0.046 *** | F _1, 29_ = 0.007  p = 0.934 |
|  | *Maob* | 34 | F _1, 30_ = 0.829  p = 0.370 | F _1, 30_ = 1.103  p = 0.302 | F _1, 30_ = 0.916  p = 0.346 |
|  | *Slc1a2* | 34 | F _1, 30_ = 0.522  p = 0.476 | F _1, 30_ = 0.001  p = 0.974 | F _1, 30_ = 0.111  p = 0.742 |
|  | *Slc6a3* |  | - | | |
|  | ***Slc6a4*** | 32 | F _1, 28_ = 0.443  p = 0.511 | F _1, 28_ = 0.134  p = 0.717 | **F _1, 28_ = 5.254**  **p = 0.030 *** |
|  | *Th* | 34 | F _1, 30_ = 1.870  p = 0.182 | F _1, 30_ = 1.025  p = 0.319 | F _1, 30_ = 0.302  p = 0.587 |
|  | *Tph2* | 34 | F _1, 30_ = 0.428  p = 0.518 | F _1, 30_ = 1.932  p = 0.175 | F _1, 30_ = 0.083  p = 0.776 |
| **mPFC** | ***Adh1*** | 34 | **Kruskal-Wallis test: p = 0.033 *** | | |
|  | *Cat* | 34 | F _1, 30_ = 0.010  p = 0.922 | F _1, 30_ = 2.654  p = 0.114 | F _1, 30_ = 0.884  p = 0.355 |
|  | ***Comt*** | 34 | F _1, 30_ = 0.854  p = 0.363 | **F _1, 30_ = 13.27**  **p = 0.001 **** | F _1, 30_ = 1.816  p = 0.188 |
|  | *Drd1* | 34 | F _1, 30_ = 1.053  p = 0.313 | F _1, 30_ = 2.524  p = 0.123 | F _1, 30_ = 0.193  p = 0.664 |
|  | *Drd2* | 34 | F _1, 30_ = 3.976  p = 0.055 | F _1, 30_ = 0.003  p = 0.958 | F _1, 30_ = 0.008  p = 0.929 |
|  | *Gabbr1* | 34 | F _1, 30_ = 0.016  p = 0.900 | F _1, 30_ = 0.399  p = 0.533 | F _1, 30_ = 0.004  p = 0.950 |
|  | *Gabbr2* | 33 | F _1, 29_ = 0.090  p = 0.766 | F _1, 29_ = 1.590  p = 0.693 | F _1, 29_ = 0.002  p = 0.963 |
|  | *Gabra1* | 34 | Kruskal-Wallis test: p = 0.817 | | |
|  | *Gad1* | 34 | F _1, 30_ = 1.421  p = 0.243 | F _1, 30_ = 1.249  p = 0.273 | F _1, 30_ = 0.058  p = 0.812 |
|  | *Gad2* | 34 | F _1, 30_ = 0.367  p = 0.549 | F _1, 30_ = 1.475  p = 0.234 | F _1, 30_ = 0.461  p = 0.502 |
|  | *Gria1* | 34 | F _1, 30_ = 0.744  p = 0.395 | F _1, 30_ = 0.522  p = 0.476 | F _1, 30_ = 0.178  p = 0.676 |
|  | *Grin2a* | 34 | F _1, 30_ = 3.586  p = 0.068 | F _1, 30_ = 0.064  p = 0.802 | F _1, 30_ = 0.264  p = 0.611 |
|  | *Grin2b* | 33 | F _1, 29_ = 1.125  p = 0.298 | F _1, 29_ = 0.965  p = 0.334 | F _1, 29_ = 0.033  p = 0.857 |
|  | *Grm2* | 34 | F _1, 30_ = 0.104  p = 0.749 | F _1, 30_ = 0.047  p = 0.831 | F _1, 30_ = 0.065  p = 0.801 |
|  | *Grm3* | 34 | F _1, 30_ = 0.913  p = 0.347 | F _1, 30_ = 0.074  p = 0.787 | F _1, 30_ = 0.151  p = 0.701 |
|  | *Grm5* | 32 | F _1, 28_ = 0.646  p = 0.429 | F _1, 28_ = 0.687  p = 0.414 | F _1, 28_ = 1.256  p = 0.272 |
|  | *Htr1a* | 34 | F _1, 30_ = 0.676  p = 0.417 | F _1, 30_ = 0.145  p = 0.706 | F _1, 30_ = 0.018  p = 0.895 |
|  | *Htr2a* | 34 | F _1, 30_ = 0.098  p = 0.756 | F _1, 30_ = 0.476  p = 0.496 | F _1, 30_ = 0.258  p = 0.615 |
|  | ***Htr2b*** | 27 | F _1, 23_ = 0.282  p = 0.601 | **F _1, 23_ = 6.437**  **p = 0.018 *** | F _1, 23_ = 0.062  p = 0.806 |
|  | *Htr3a* | 34 | F _1, 30_ = 3.179  p = 0.085 | F _1, 30_ = 0.382  p = 0.541 | F _1, 30_ = 0.022  p = 0.883 |
|  | *Npy* | 34 | F _1, 30_ = 0.280  p = 0.601 | F _1, 30_ = 1.708  p = 0.201 | F _1, 30_ = 1.889  p = 0.180 |
|  | ***Maoa*** | 34 | F _1, 30_ = 1.061  p = 0.311 | F _1, 30_ = 0.427  p = 0.518 | **F _1, 30_ = 5.229**  **p = 0.029 *** |
|  | *Maob* | 34 | F _1, 30_ = 0.303  p = 0.587 | F _1, 30_ = 2.103  p = 0.157 | F _1, 30_ = 0.373  p = 0.546 |
|  | *Slc1a2* | 34 | F _1, 30_ = 0.088  p = 0.769 | F _1, 30_ = 0.391  p = 0.537 | F _1, 30_ = 0.296  p = 0.590 |
|  | *Slc6a3* | 19 | F _1, 15_ = 0.227  p = 0.640 | F _1, 15_ = 0.064  p = 0.804 | F _1, 15_ = 0.656  p = 0.431 |
|  | *Slc6a4* | 34 | F _1, 30_ = 0.076  p = 0.785 | F _1, 30_ = 0.026  p = 0.873 | F _1, 30_ = 2.704  p = 0.111 |
|  | *Th* | 34 | F _1, 30_ = 0.001  p = 0.977 | F _1, 30_ = 0.375  p = 0.545 | F _1, 30_ = 0.197  p = 0.661 |
|  | *Tph2* | 33 | F _1, 29_ = 0.0003  p = 0.985 | F _1, 29_ = 1.301  p = 0.263 | F _1, 29_ = 2.364  p = 0.135 |
| **Amy** | *Adh1* | 31 | F _1, 27_ = 1.335  p = 0.258 | F _1, 27_ = 2.556  p = 0.122 | F _1, 27_ = 4.158  p = 0.051 |
|  | *Cat* | 31 | F _1, 27_ = 0.535  p = 0.471 | F _1, 27_ = 0.564  p = 0.459 | F _1, 27_ = 0.095  p = 0.761 |
|  | *Comt* | 32 | F _1, 28_ = 0.069  p = 0.795 | F _1, 28_ = 0.366  p = 0.550 | F _1, 28_ = 1.749  p = 0.197 |
|  | *Drd1* | 32 | F _1, 28_ = 3.270  p = 0.081 | F _1, 28_ = 0.054  p = 0.818 | F _1, 28_ = 2.768  p = 0.107 |
|  | ***Drd2*** | 32 | F _1, 28_ = 1.579  p = 0.219 | **F _1, 28_ = 4.436**  **p = 0.044 *** | F _1, 28_ = 0.034  p = 0.856 |
|  | *Gabbr1* | 31 | F _1, 27_ = 0.054  p = 0.818 | F _1, 27_ = 0.483  p = 0.493 | F _1, 27_ = 3.117  p = 0.089 |
|  | *Gabbr2* | 32 | F _1, 28_ = 0.248  p = 0.622 | F _1, 28_ = 0.862  p = 0.361 | F _1, 28_ = 0.386  p = 0.539 |
|  | *Gabra1* | 32 | F _1, 28_ = 0.001  p = 0.973 | F _1, 28_ = 4.136 x 10^-6^  p = 0.998 | F _1, 28_ = 0.260  p = 0.614 |
|  | *Gad1* | 32 | F _1, 28_ = 1.069  p = 0.310 | F _1, 28_ = 2.542  p = 0.122 | F _1, 28_ = 0.562  p = 0.460 |
|  | *Gad2* | 32 | Kruskal-Wallis test: p = 0.190 | | |
|  | *Gria1* | 32 | F _1, 28_ = 0.010  p = 0.921 | F _1, 28_ = 0.039  p = 0.845 | F _1, 28_ = 0.172  p = 0.682 |
|  | *Grin2a* | 32 | F _1, 28_ = 0.200  p = 0.658 | F _1, 28_ = 1.348  p = 0.255 | F _1, 28_ = 0.077  p = 0.783 |
|  | *Grin2b* | 32 | F _1, 28_ = 1.567  p = 0.221 | F _1, 28_ = 0.001  p = 0.973 | F _1, 28_ = 0.434  p = 0.516 |
|  | *Grm2* | 32 | F _1, 28_ = 0.439  p = 0.513 | F _1, 28_ = 0.928  p = 0.343 | F _1, 28_ = 0.062  p = 0.805 |
|  | *Grm3* | 32 | F _1, 28_ = 0.012  p = 0.911 | F _1, 28_ = 0.406  p = 0.529 | F _1, 28_ = 0.786  p = 0.383 |
|  | *Grm5* | 32 | F _1, 28_ = 3.764  p = 0.063 | F _1, 28_ = 2.298  p = 0.141 | F _1, 28_ = 1.017  p = 0.322 |
|  | *Htr1a* | 32 | F _1, 28_ = 1.339  p = 0.257 | F _1, 28_ = 3.505  p = 0.072 | F _1, 28_ = 0.177  p = 0.678 |
|  | *Htr2a* | 32 | F _1, 28_ = 0.005  p = 0.942 | F _1, 28_ = 0.040  p = 0.844 | F _1, 28_ = 1.719  p = 0.201 |
|  | *Htr2b* | 26 | F _1, 22_ = 2.124  p = 0.159 | F_1, 22_ = 1.577  p = 0.222 | F_1, 22_ = 1.334  p = 0.261 |
|  | *Htr3a* | 32 | F _1, 28_ = 0.484  p = 0.492 | F _1, 28_ = 3.476  p = 0.073 | F _1, 28_ = 0.637  p = 0.432 |
|  | *Npy* | 32 | F _1, 28_ = 0.001  p = 0.970 | F _1, 28_ = 0.037  p = 0.849 | F _1, 28_ = 0.778  p = 0.385 |
|  | *Maoa* | 31 | F _1, 27_ = 0.263  p = 0.612 | F _1, 27_ = 0.483  p = 0.493 | F _1, 27_ = 3.117  p = 0.089 |
|  | *Maob* | 32 | F _1, 28_ = 3.812 x 10^-5^  p = 0.995 | F _1, 28_ = 0.148  p = 0.703 | F _1, 28_ = 0.924  p = 0.345 |
|  | *Slc1a2* | 32 | F _1, 28_ = 0.021  p = 0.885 | F _1, 28_ = 1.374  p = 0.350 | F _1, 28_ = 0.902  p = 0.350 |
|  | *Slc6a3* | 24 | F _1, 20_ = 0.383  p = 0.543 | F _1, 20_ = 3.661  p = 0.070 | F _1, 20_ = 3.568 x 10^-8^  p = 0.999 |
|  | *Slc6a4* | 32 | F _1, 28_ = 0.330  p = 0.570 | F _1, 28_ = 0.022  p = 0.884 | F _1, 28_ = 0.367  p = 0.550 |
|  | *Th* | 32 | F _1, 28_ = 2.016  p = 0.167 | F _1, 28_ = 1.813  p = 0.189 | F _1, 28_ = 0.805  p = 0.377 |
|  | *Tph2* | 32 | F _1, 28_ = 2.02 x 10^-5^  p = 0.996 | F _1, 28_ = 2.390  p = 0.133 | F _1, 28_ = 0.301  p = 0.588 |
| **OFC** | *Adh1* | 34 | Kruskal-Wallis test: p = 0.967 | | |
|  | *Cat* | 34 | F _1, 30_ = 0.187  p = 0.669 | F _1, 30_ = 3.842  p = 0.059 | F _1, 30_ = 1.268  p = 0.269 |
|  | *Comt* | 34 | F _1, 30_ = 0.0004  p = 0.984 | F _1, 30_ = 0.771  p = 0.387 | F _1, 30_ = 3.034  p = 0.092 |
|  | *Drd1* | 34 | F _1, 30_ = 0.023  p = 0.880 | F _1, 30_ = 3.916  p = 0.057 | F _1, 30_ = 0.081  p = 0.777 |
|  | *Drd2* | 32 | F _1, 28_ = 0.249  p = 0.622 | F _1, 28_ = 0.352  p = 0.558 | F _1, 28_ = 0.169  p = 0.684 |
|  | *Gabbr1* | 34 | F _1, 30_ = 0.180  p = 0.674 | F _1, 30_ = 0.284  p = 0.598 | F _1, 30_ = 1.273  p = 0.268 |
|  | *Gabbr2* | 34 | F _1, 30_ = 0.003  p = 0.954 | F _1, 30_ = 2.157  p = 0.152 | F _1, 30_ = 0.079  p = 0.780 |
|  | *Gabra1* | 34 | F _1, 30_ = 0.523  p = 0.475 | F _1, 30_ = 0.006  p = 0.938 | F _1, 30_ = 1.166  p = 0.289 |
|  | *Gad1* | 34 | F _1, 30_ = 0.275  p = 0.604 | F _1, 30_ = 2.158  p = 0.152 | F _1, 30_ = 0.171  p = 0.683 |
|  | ***Gad2*** | 34 | F _1, 30_ = 6.23 x 10^-6^  p = 0.998 | **F _1, 30_ = 4.390**  **p =** **0.045 *** | F _1, 30_ = 0.337  p = 0.566 |
|  | ***Gria1*** | 34 | F _1, 30_ = 0.236  p = 0.631 | F _1, 30_ = 0.191  p = 0.665 | **F _1, 30_ = 6.268**  **p = 0.018 *** |
|  | ***Grin2a*** | 34 | **F _1, 30_ = 4.629**  **p = 0.040 *** | F _1, 30_ = 0.492  p = 0.488 | F _1, 30_ = 0.036  p = 0.851 |
|  | *Grin2b* | 34 | F _1, 30_ = 0.032  p = 0.860 | F _1, 30_ = 0.898  p = 0.351 | F _1, 30_ = 0.881  p = 0.356 |
|  | *Grm2* | 34 | F _1, 30_ = 0.427  p = 0.518 | F _1, 30_ = 0.006  p = 0.937 | F _1, 30_ = 0.948  p = 0.338 |
|  | *Grm3* | 34 | F _1, 30_ = 0.236  p = 0.631 | F _1, 30_ = 0.014  p = 0.907 | F _1, 30_ = 2.046  p = 0.163 |
|  | *Grm5* | 34 | F _1, 30_ = 1.221  p = 0.278 | F _1, 30_ = 1.933  p = 0.175 | F _1, 30_ = 0.023  p = 0.882 |
|  | ***Htr1a*** | 34 | F _1, 30_ = 0.689  p = 0.413 | **F _1, 30_ = 8.506**  **p =** **0.007 **** | F _1, 30_ = 0.467  p = 0.450 |
|  | *Htr2a* | 34 | F _1, 30_ = 0.080  p = 0.780 | F _1, 30_ = 1.440  p = 0.240 | F _1, 30_ = 0.825  p = 0.371 |
|  | *Htr2b* | 29 | F _1, 25_ = 0.102  p = 0.753 | F _1, 25_ = 4.067  p = 0.055 | F _1, 25_ = 0.804  p = 0.378 |
|  | ***Htr3a*** | 34 | F _1, 30_ = 3.393  p = 0.057 | F _1, 30_ = 2.905  p = 0.099 | **F _1, 30_ = 6.514**  **p = 0.016 *** |
|  | *Npy* | 34 | Kruskal-Wallis test: p = 0.898 | | |
|  | ***Maoa*** | 33 | F _1, 29_ = 2.643  p = 0.115 | F _1, 29_ = 3.321  p = 0.079 | **F _1, 29_ = 4.734**  **p = 0.038 *** |
|  | *Maob* | 34 | F _1, 30_ = 0.756  p = 0.392 | F _1, 30_ = 0.157  p = 0.695 | F _1, 30_ = 1.749  p = 0.196 |
|  | *Slc1a2* | 34 | F _1, 30_ = 0.682  p = 0.415 | F _1, 30_ = 0.568  p = 0.457 | F _1, 30_ = 0.009  p = 0.926 |
|  | *Slc6a3* | 33 | F _1, 29_ = 0.747  p = 0.395 | F _1, 29_ = 0.099  p = 0.755 | F _1, 29_ = 1.854  p = 0.184 |
|  | *Slc6a4* | 34 | F _1, 30_ = 0.968  p = 0.333 | F _1, 30_ = 2.197  p = 0.149 | F _1, 30_ = 0.841  p = 0.367 |
|  | *Th* | 34 | F _1, 30_ = 0.741  p = 0.396 | F _1, 30_ = 0.823  p = 0.372 | F _1, 30_ = 3.450  p = 0.073 |
|  | *Tph2* | 34 | F _1, 30_ = 0.244  p = 0.625 | F _1, 30_ = 0.014  p = 0.906 | F _1, 30_ = 0.071  p = 0.792 |
| **Nacc** | ***Adh1*** | 31 | F _1, 27_ = 1.126  p = 0.298 | **F _1, 27_ = 9.895**  **p =** **0.004 **** | F _1, 27_ = 1.793  p = 0.192 |
|  | *Cat* | 34 | F _1, 30_ = 0.409  p = 0.528 | F _1, 30_ = 0.044  p = 0.835 | F _1, 30_ = 0.257  p = 0.616 |
|  | *Comt* | 34 | F _1, 30_ = 1.301  p = 0.263 | F _1, 30_ = 1.301  p = 0.263 | F _1, 30_ = 2.589  p = 0.118 |
|  | *Drd1* | 34 | F _1, 30_ = 0.017  p = 0.898 | F _1, 30_ = 0.011  p = 0.916 | F _1, 30_ = 1.127  p = 0.297 |
|  | *Drd2* | 34 | F _1, 30_ = 0.839  p = 0.832 | F _1, 30_ = 0.297  p = 0.590 | F _1, 30_ = 0.478  p = 0.495 |
|  | *Gabbr1* | 34 | F _1, 30_ = 0.080  p = 0.780 | F _1, 30_ = 0.002  p = 0.968 | F _1, 30_ = 0.474  p = 0.497 |
|  | *Gabbr2* | 34 | Kruskal-Wallis test: p = 0.800 | | |
|  | *Gabra1* | 34 | F _1, 30_ = 0.051  p = 0.822 | F _1, 30_ = 0.152  p = 0.699 | F _1, 30_ = 0.018  p = 0.895 |
|  | *Gad1* | 34 | F _1, 30_ = 0.088  p = 0.769 | F _1, 30_ = 0.303  p = 0.586 | F _1, 30_ = 0.475  p = 0.496 |
|  | *Gad2* | 34 | F _1, 30_ = 0.277  p = 0.603 | F _1, 30_ = 1.223  p = 0.278 | F _1, 30_ = 1.425  p = 0.242 |
|  | *Gria1* | 34 | F _1, 30_ = 0.245  p = 0.624 | F _1, 30_ = 0.064  p = 0.802 | F _1, 30_ = 0.004  p = 0.948 |
|  | *Grin2a* | 34 | F _1, 30_ = 0.012  p = 0.913 | F _1, 30_ = 0.042  p = 0.840 | F _1, 30_ = 3.122  p = 0.087 |
|  | *Grin2b* | 34 | F _1, 30_ = 0.130  p = 0.721 | F _1, 30_ = 0.612  p = 0.440 | F _1, 30_ = 0.450  p = 0.508 |
|  | *Grm2* | 32 | F _1, 28_ = 1.289  p = 0.266 | F _1, 28_ = 0.167  p = 0.686 | F _1, 28_ = 0.012  p = 0.915 |
|  | *Grm3* | 34 | F _1, 30_ = 0.481  p = 0.494 | F _1, 30_ = 0.001  p = 0.977 | F _1, 30_ = 0.344  p = 0.562 |
|  | *Grm5* | 34 | F _1, 30_ = 0.018  p = 0.893 | F _1, 30_ = 0.447  p = 0.509 | F _1, 30_ = 0.098  p = 0.756 |
|  | *Htr1a* | 34 | F _1, 30_ = 0.040  p = 0.843 | F _1, 30_ = 0.315  p = 0.579 | F _1, 30_ = 2.250  p = 0.144 |
|  | *Htr2a* | 34 | F _1, 30_ = 0.981  p = 0.330 | F _1, 30_ = 1.415  p = 0.244 | F _1, 30_ = 0.009  p = 0.923 |
|  | *Htr2b* | 23 | F _1, 19_ = 0.011  p = 0.918 | F _1, 19_ = 0.693  p = 0.416 | F _1, 19_ = 3.959  p = 0.061 |
|  | *Htr3a* | 34 | F _1, 30_ = 2.149  p = 0.153 | F _1, 30_ = 0.051  p = 0.823 | F _1, 30_ = 0.029  p = 0.866 |
|  | *Npy* | 34 | F _1, 30_ = 0.460  p = 0.503 | F _1, 30_ = 0.051  p = 0.823 | F _1, 30_ = 0.097  p = 0.758 |
|  | *Maoa* | 34 | F _1, 30_ = 0.243  p = 0.626 | F _1, 30_ = 0.594  p = 0.447 | F _1, 30_ = 1.694  p = 0.203 |
|  | *Maob* | 34 | F _1, 30_ = 1.089  p = 0.305 | F _1, 30_ = 0.756  p = 0.392 | F _1, 30_ = 0.554  p = 0.462 |
|  | *Slc1a2* | 34 | F _1, 30_ = 0.707  p = 0.407 | F _1, 30_ = 0.046  p = 0.832 | F _1, 30_ = 1.002  p = 0.325 |
|  | *Slc6a3* | 34 | F _1, 30_ = 0.143  p = 0.708 | F _1, 30_ = 3.698  p = 0.064 | F _1, 30_ = 1.647  p = 0.209 |
|  | ***Slc6a4*** | 33 | F _1, 29_ = 0.027  p = 0.872 | **F _1, 29_ = 5.258**  **p = 0.029 *** | F _1, 29_ = 2.349  p = 0.136 |
|  | *Th* | 33 | F _1, 29_ = 0.895  p = 0.352 | F _1, 29_ = 0.699  p = 0.410 | F _1, 29_ = 3.685  p = 0.065 |
|  | *Tph2* | 34 | F _1, 30_ = 3.384  p = 0.076 | F _1, 30_ = 0.004  p = 0.952 | F _1, 30_ = 0.002  p = 0.963 |

**Table S2. The effects of trait sensitivity to NF and alcohol drinking on protein level.**

Statistically significant effects and interactions are bolded (2-way ANOVA).

| **Structure** | **Protein** | ***n*** | ***Interaction*** | ***Treatment*** | ***Sensitivity*** |
| --- | --- | --- | --- | --- | --- |
| **ACC** | SERT | 34 | F_1, 30_ = 0.992  p = 0.327 | F_1, 30_ = 0.009  p = 0.925 | F_1, 30_ = 0.424  p = 0.520 |
| **mPFC** | **ADH1** | 34 | F_1, 30_ = 0.136  p = 0.715 | **F_1, 30_ = 13.28**  **p = 0.001 **** | F_1, 30_ = 0.043  p = 0.836 |
|  | **MAO-A** | 33 | **F_1, 29_ = 8.168**  **p = 0.008 **** | F_1, 29_ = 1.135  p = 0.296 | F_1, 29_ = 1.532  p = 0.226 |
| **OFC** | MAO-A | 34 | F_1, 30_ = 0.344  p = 0.562 | F_1, 30_ = 0.787  p = 0.382 | F_1, 30_ = 0.251  p = 0.620 |
|  | 5-HT3A | 33 | F_1, 29_ = 0.343  p = 0.563 | F_1, 29_ = 2.915  p = 0.099 | F_1, 29_ = 0.055  p = 0.816 |
| **Nacc** | **ADH1** | 32 | F_1, 28_ = 1.022  p = 0.321 | **F_1, 28_ = 10.38**  **p = 0.003 **** | F_1, 28_ = 1.304  p = 0.263 |
|  | SERT | 28 | F_1, 24_ = 0. 086  p = 0.772 | F_1, 24_ = 3.485  p = 0.074 | F_1, 24_ = 0.004  p = 0.951 |
