# Supplementary material for: Identification of genes regulated by trait sensitivity to negative feedback and prolonged alcohol consumption in rats
Source: Pharmacol Rep. 2024 Jan 3;76(1):207–15. doi: 10.1007/s43440-023-00563-4 (PMC10830829; doi:10.1007/s43440-023-00563-4)
Supplement: Supplementary file 3 — Supplementary file3 (DOCX 10555 KB) [file 43440_2023_563_MOESM3_ESM.docx]

**Original Western blot images used for quantification of protein levels.**

Some protein bands were unsuitable for quantification due to technical errors and these are indicated by black frames

|  | EtOH | | | | | | | H_2_O | | | | | | |
| --- | --- | --- | --- | --- | --- | --- | --- | --- | --- | --- | --- | --- | --- | --- |
| Membrane 1 | NFL | NFL | NFL | NFL | NFM | NFM | NFM | NFM | NFL | NFL | NFL | NFM | NFM | NFM |
| Rat’s number | 3 | 7 | 1 | 2 | 17 | 4 | 12 | 18 | 13 | 15 | 20 | 10 | 25 | 26 |
| Membrane 2 | NFL | NFL | NFL | NFL | NFM | NFM | NFM | NFM | NFM | NFM | NFM | NFL | NFL | NFL |
| Rat’s number | 2 | 5 | 9 | 29 | 17 | 19 | 16 | 22 | 28 | 36 | 30 | 24 | 38 | 39 |
| Membrane 3 | NFL | NFL | NFM | NFM | NFM | NFL | NFL | NFL | NFL | NFL | NFM | NFM | NFM | NFM |
| Rat’s number | 33 | 35 | 21 | 12 | 19 | 7 | 3 | 6 | 11 | 30 | 27 | 31 | 14 | 10 |

NFL – less sensitive to negative feedback

NFM – more sensitive to negative feedback















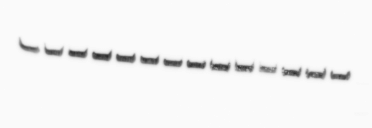

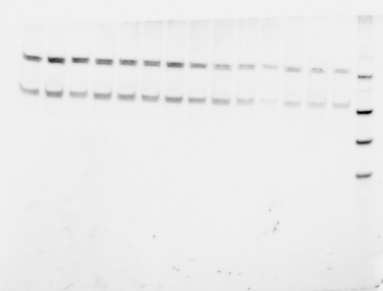


ADH1_mPFC_membrane 1

**ADH1_mPFC**


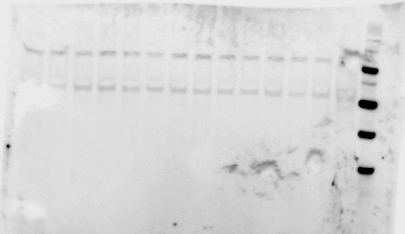


ADH1_mPFC_membrane 2


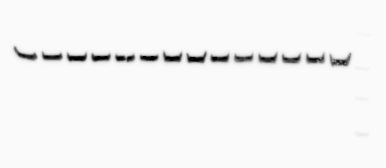

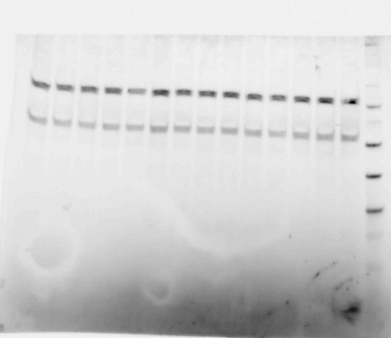


ADH1_mPFC_membrane 3


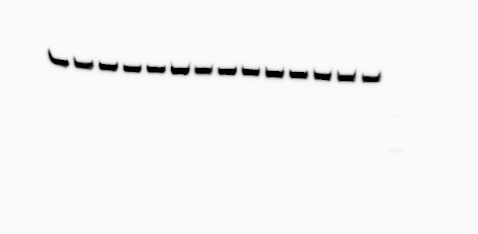


**40 kDa**

**42 kDa**

**42 kDa**

**40 kDa**

**42 kDa**

**40 kDa**

βactin

βactin

βactin

**42 kDa**

**42 kDa**

**42 kDa**

**60 kDa**

**60 kDa**

**60 kDa**

Mao-A_mPFC_membrane 2

Mao-A_mPFC_membrane 3

**Mao-A_mPFC**

βactin

Mao-A_mPFC_membrane 1

βactin

βactin

**Mao-A_OFC**


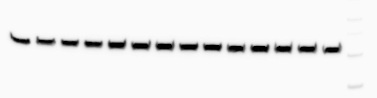


Mao-A_OFC_membrane 2

βactin


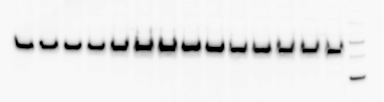

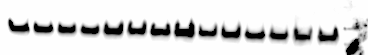

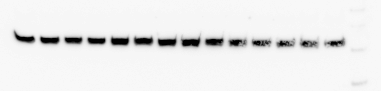


Mao-A_OFC_membrane 1

βactin


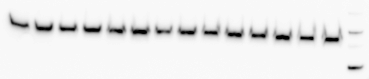


Mao-A_OFC_membrane 3


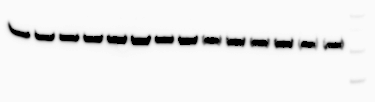


βactin

**60 kDa**

**42 kDa**

**60 kDa**

**42 kDa**

**60 kDa**

**42 kDa**


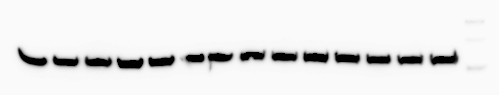

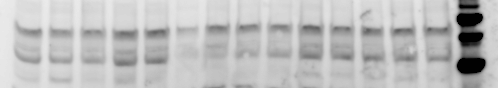

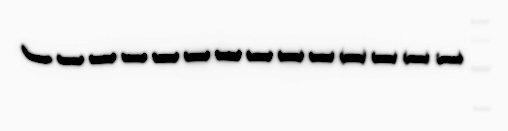

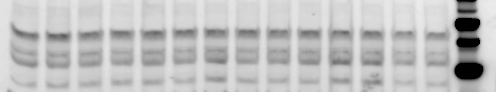

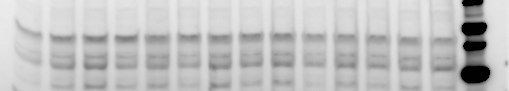


5_HT3_OFC_membrane 1

**5-HT3_OFC**

βactin

βactin


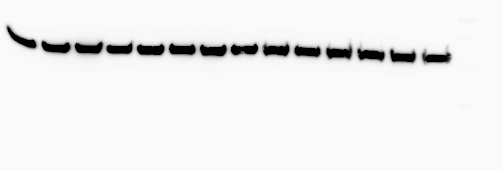


βactin

5_HT3_OFC_membrane 2

5_HT3_OFC_membrane 3

**42 kDa**

**52 kDa**

**52 kDa**

**52 kDa**

**42 kDa**

**42 kDa**

**80 kDa**

**80 kDa**

**80 kDa**

**42 kDa**

**42 kDa**

**42 kDa**


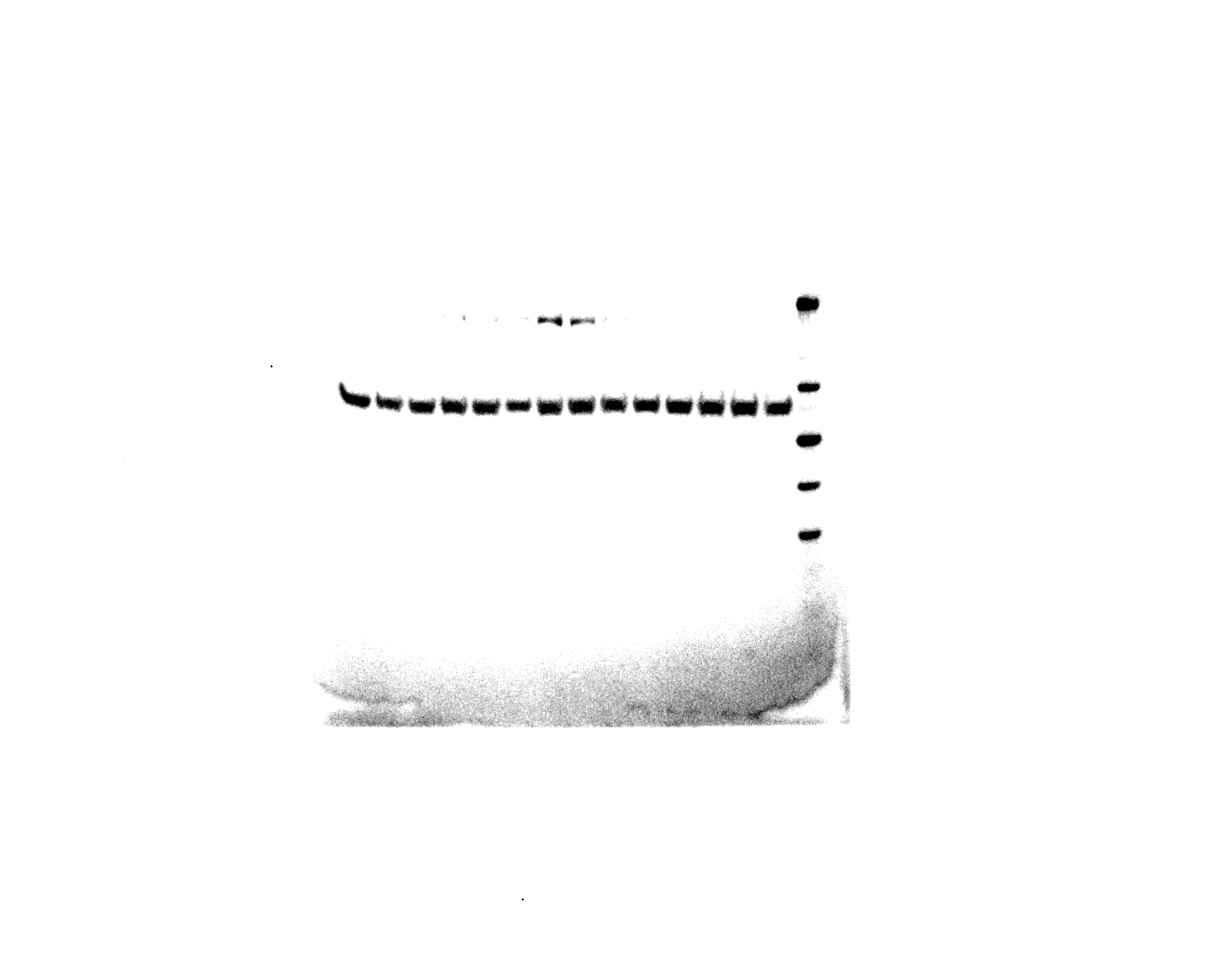


SERT_ACC_membrane 1


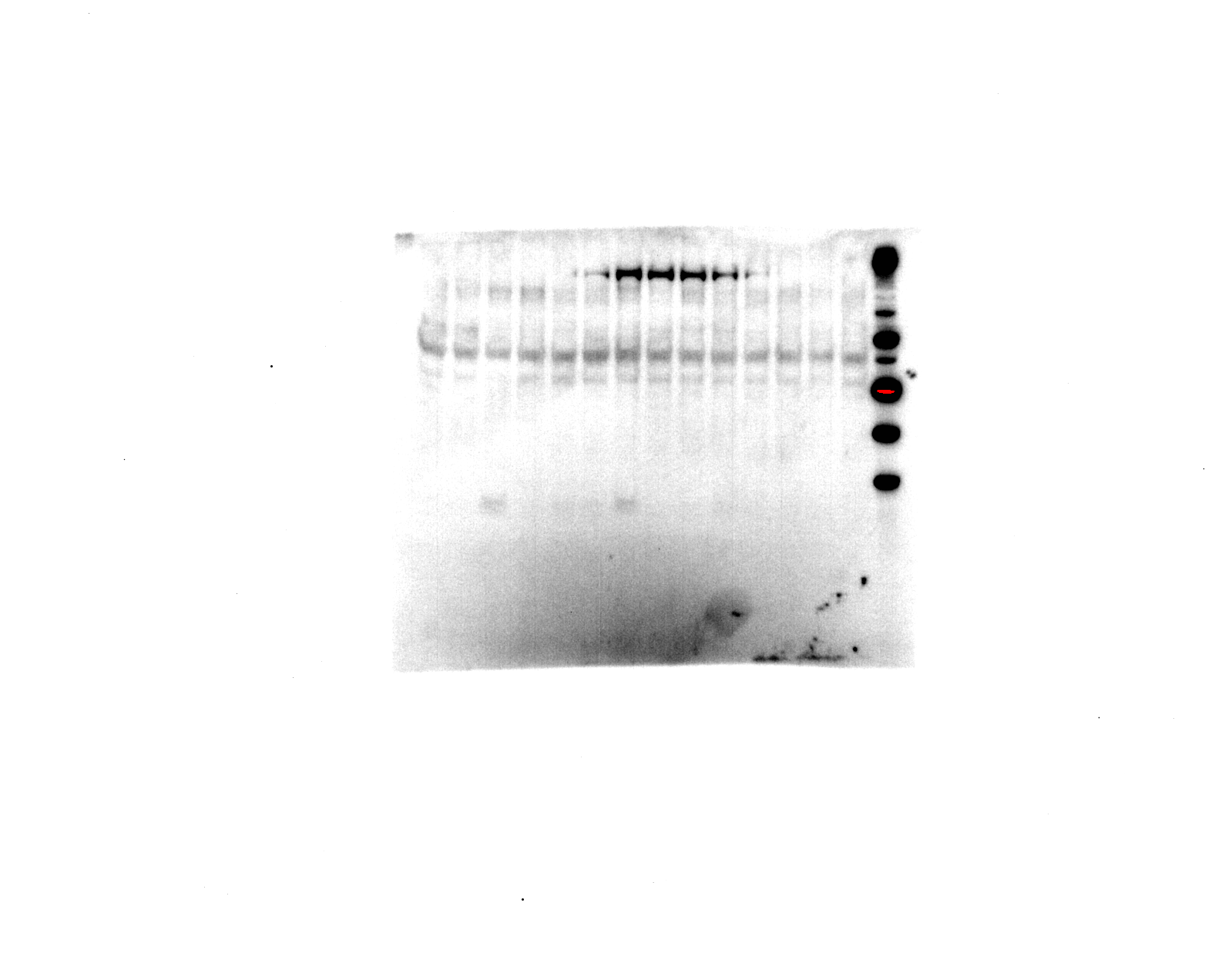


SERT_ACC_membrane 2


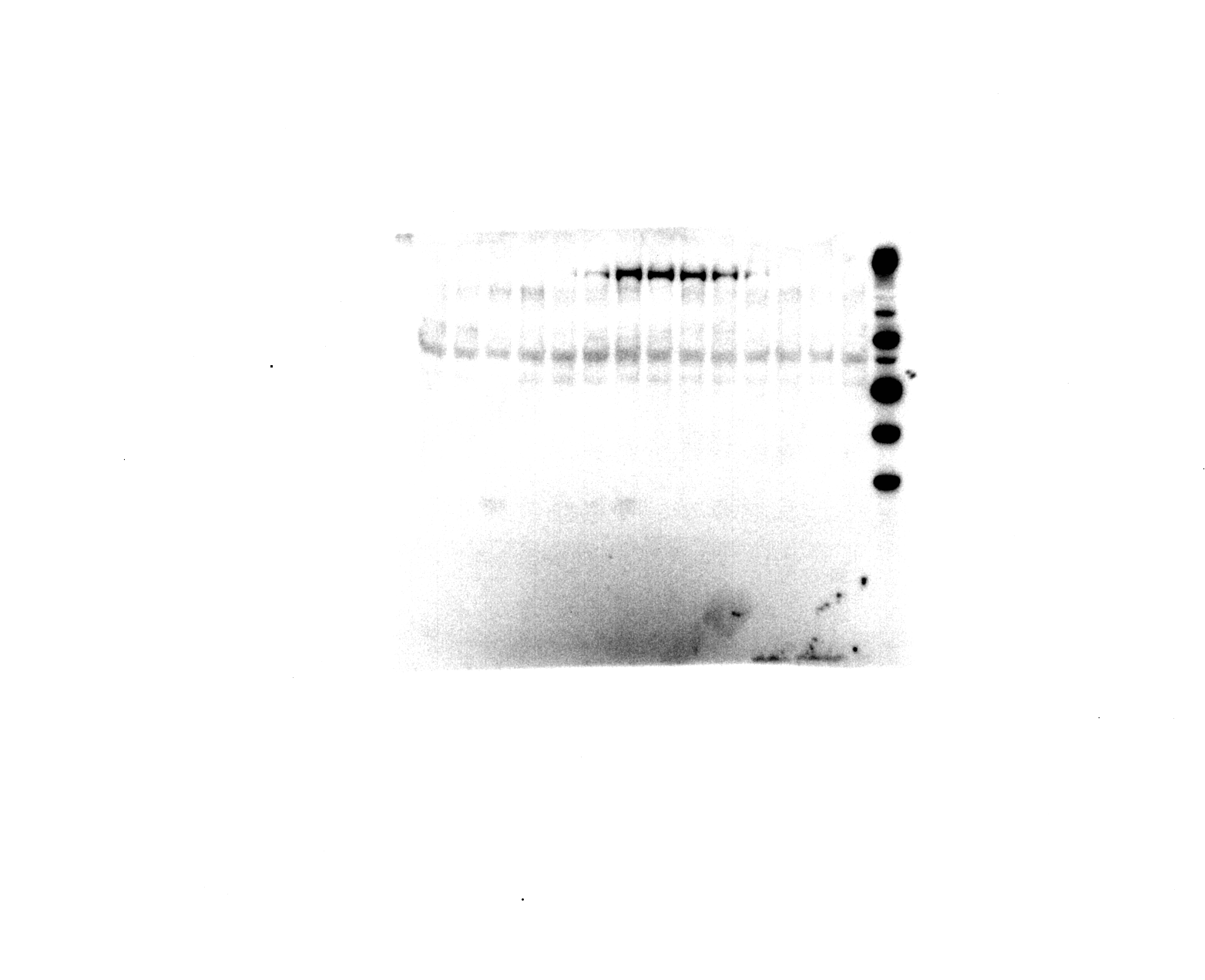


SERT_ACC_membrane 3

**SERT_ACC**


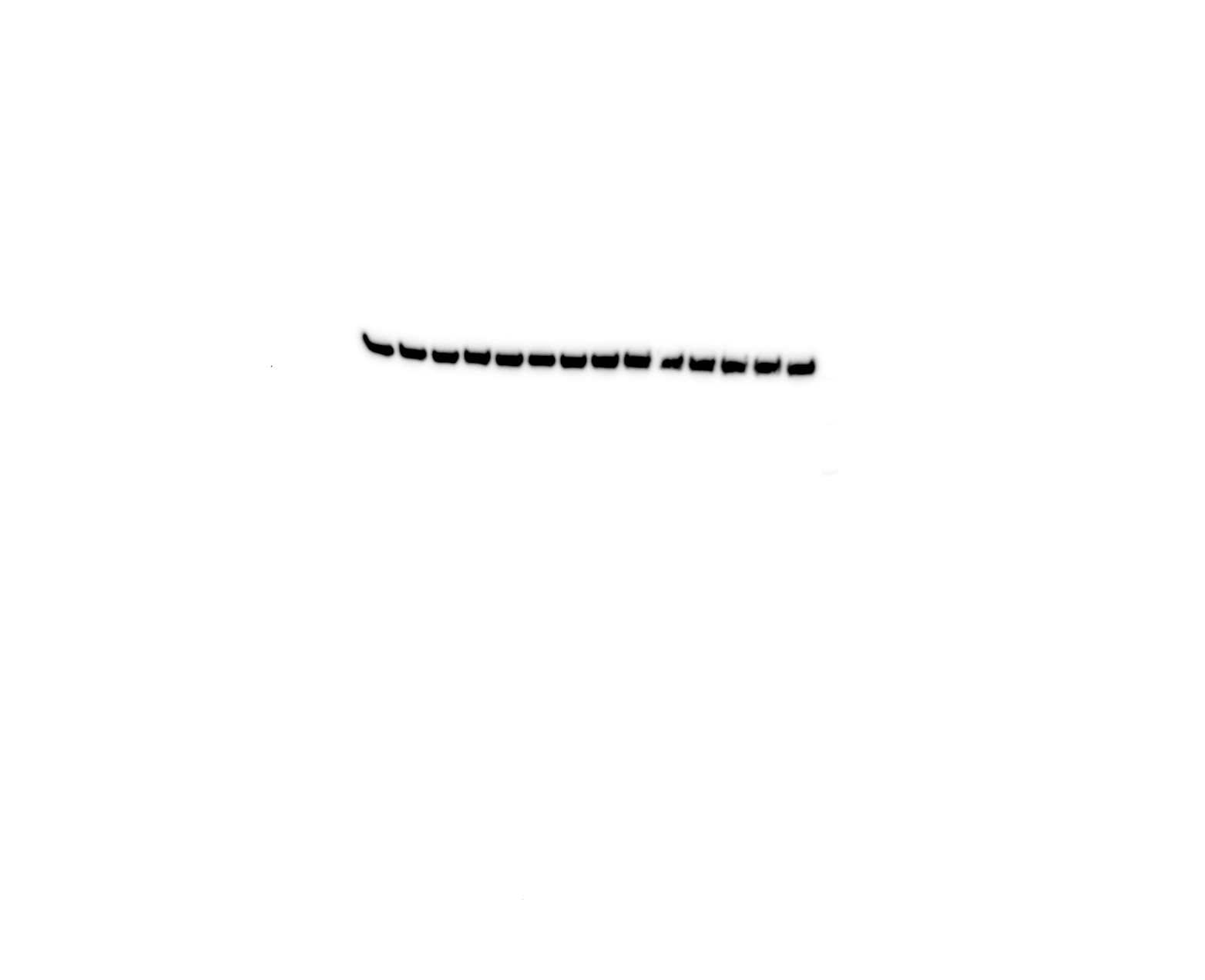


βactin


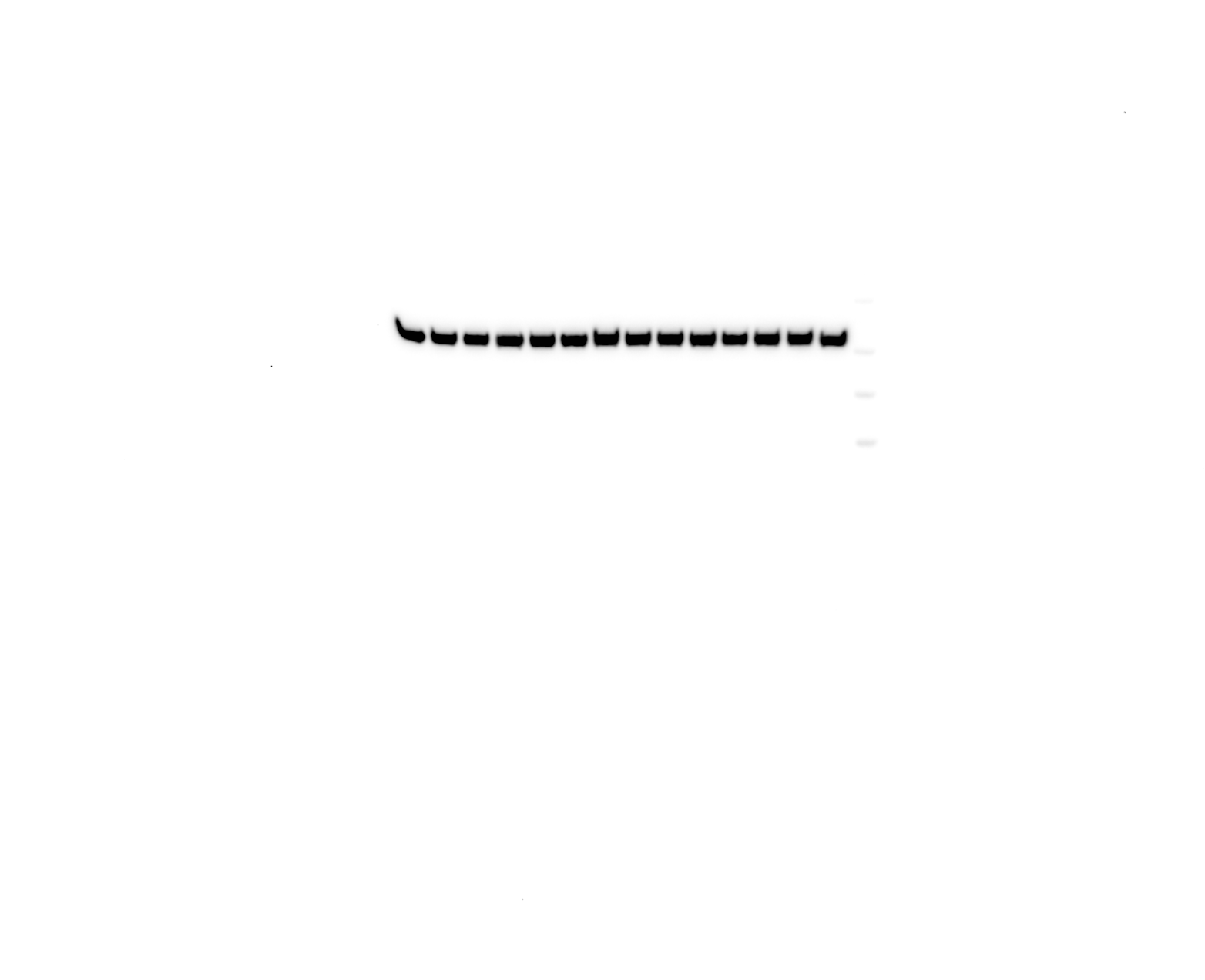


βactin


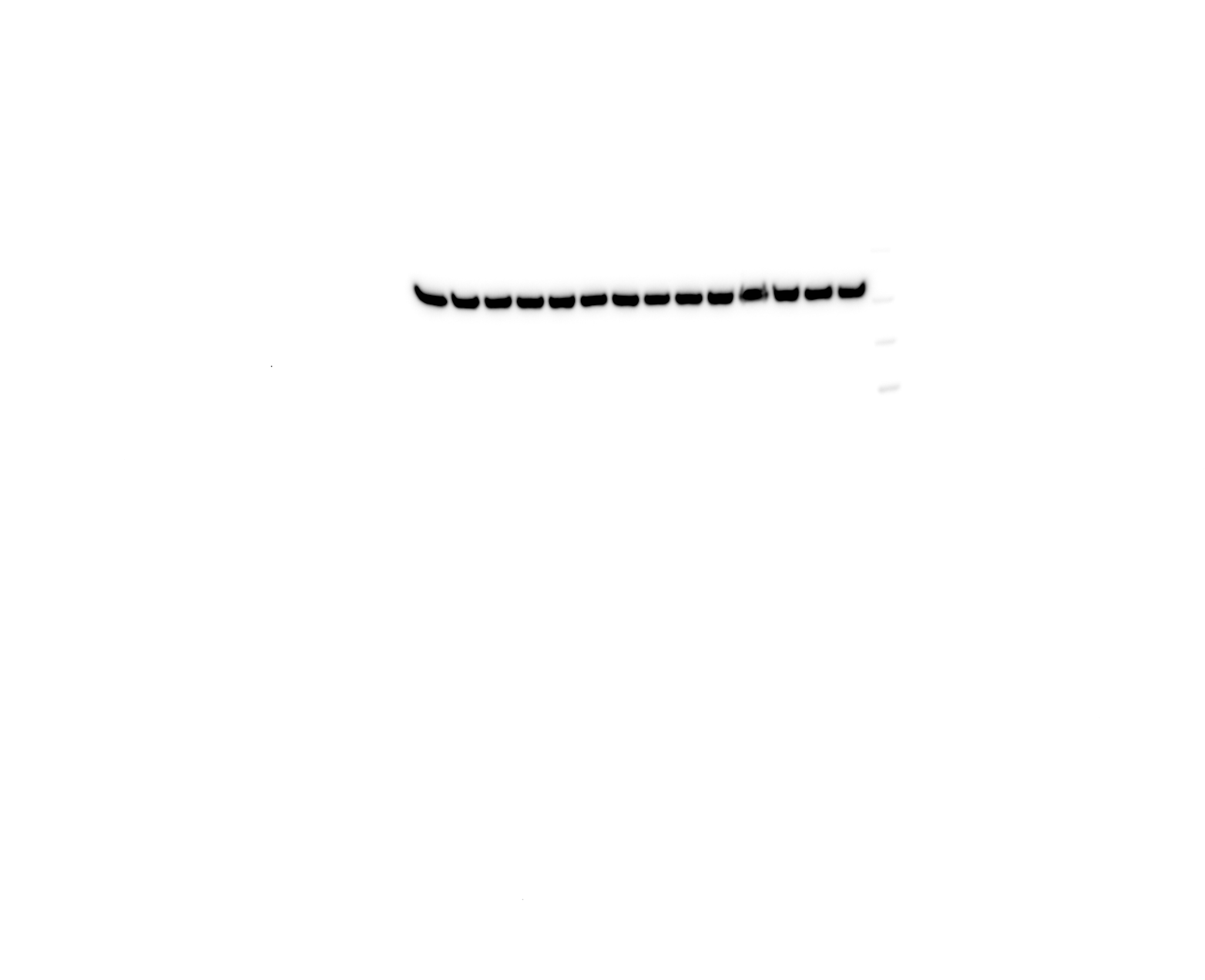


βactin

**80 kDa**

**80 kDa**

**42 kDa**

**42 kDa**


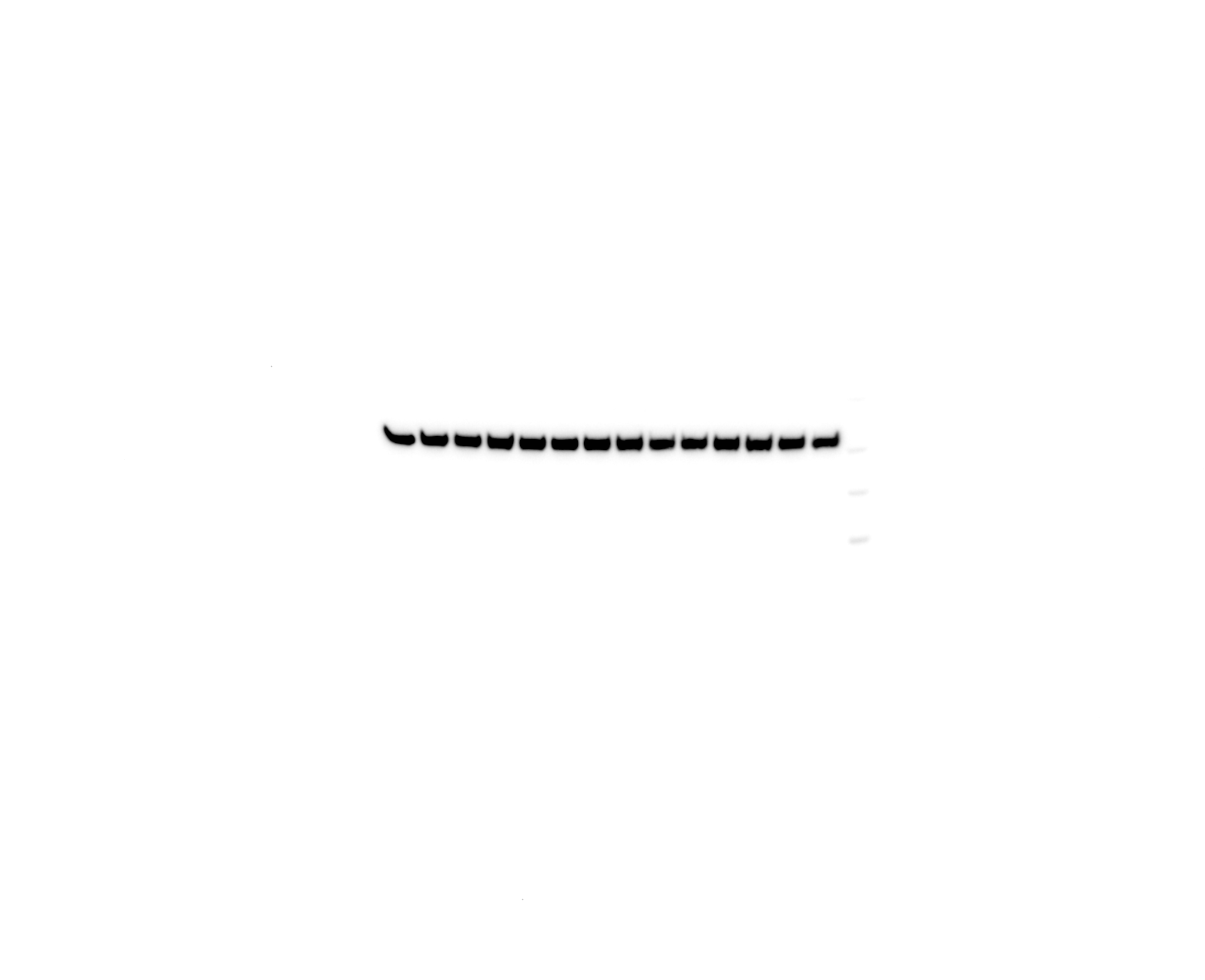

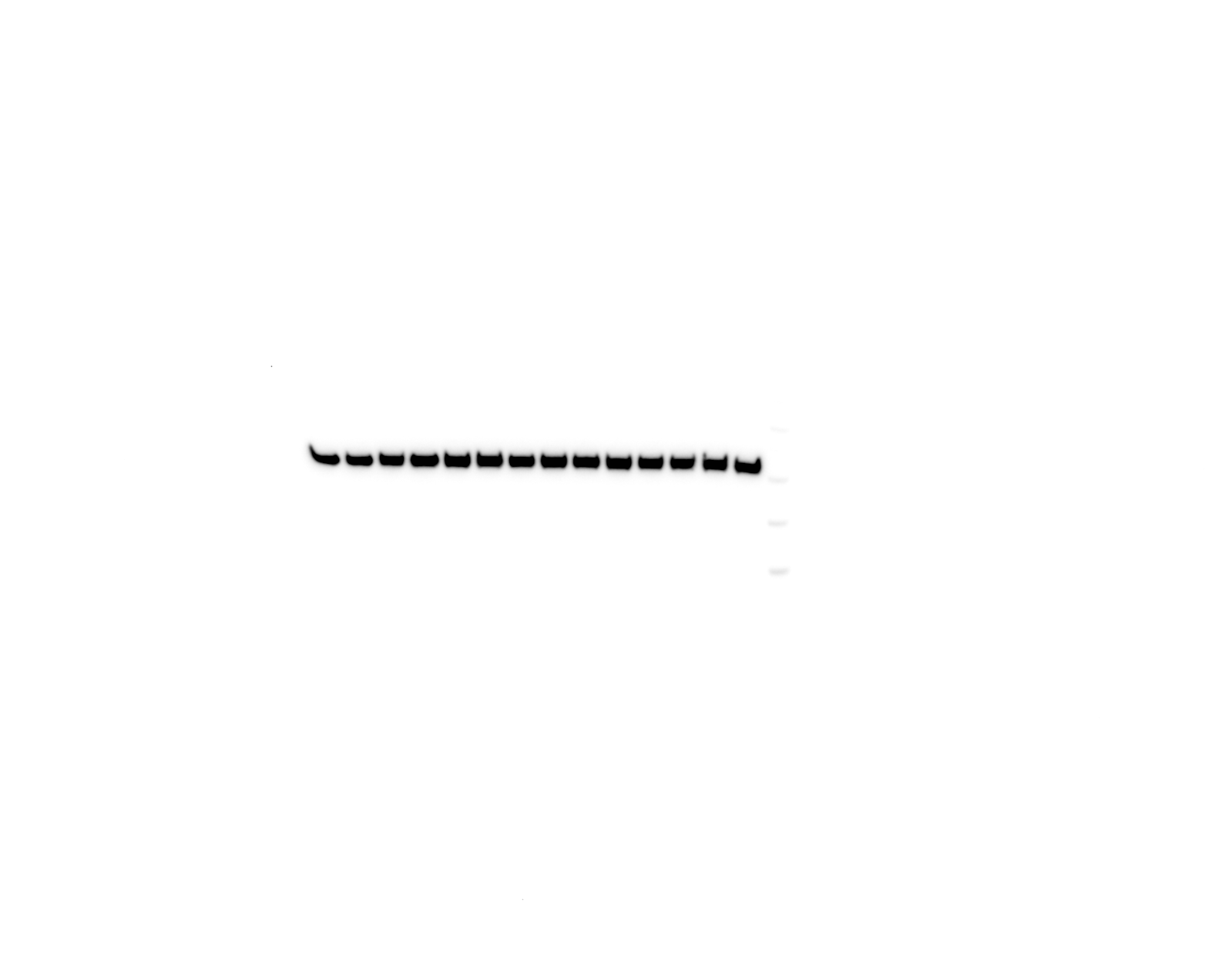


**SERT_Nacc**


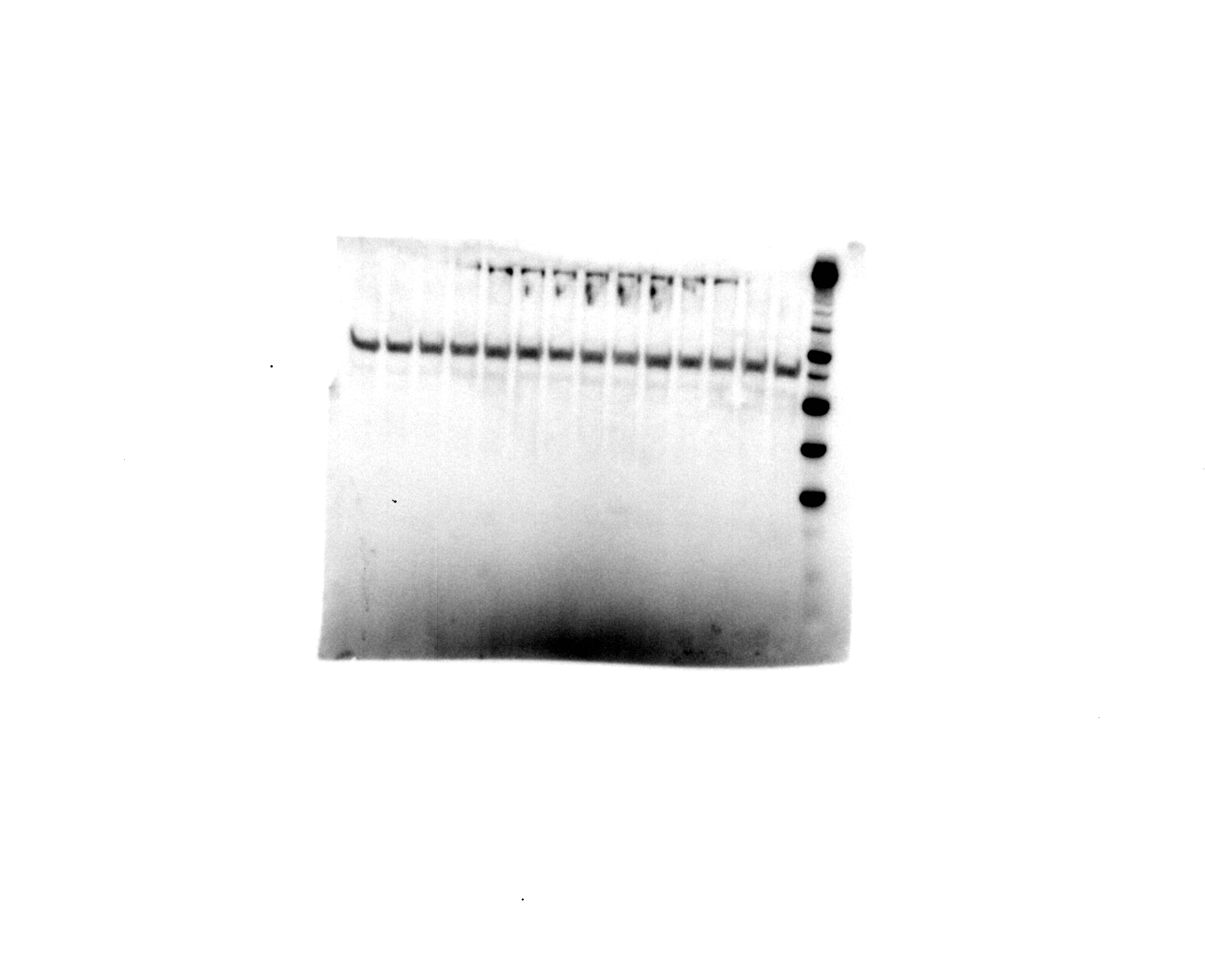

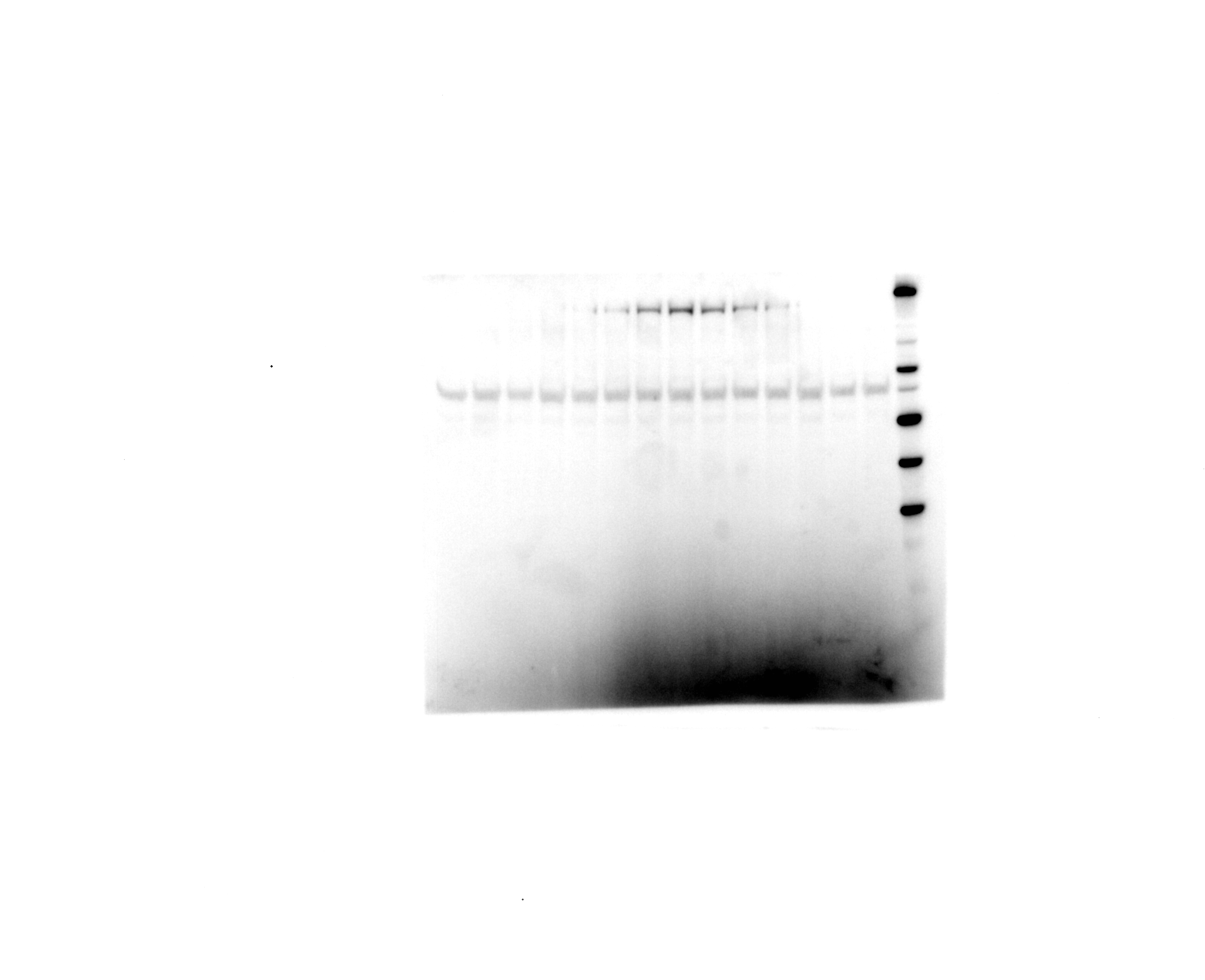


SERT_Nacc_membrane 1

SERT_Nacc_membrane 2

βactin

βactin

**40 kDa**

**40 kDa**

**40 kDa**

**42 kDa**

**42 kDa**

**42 kDa**


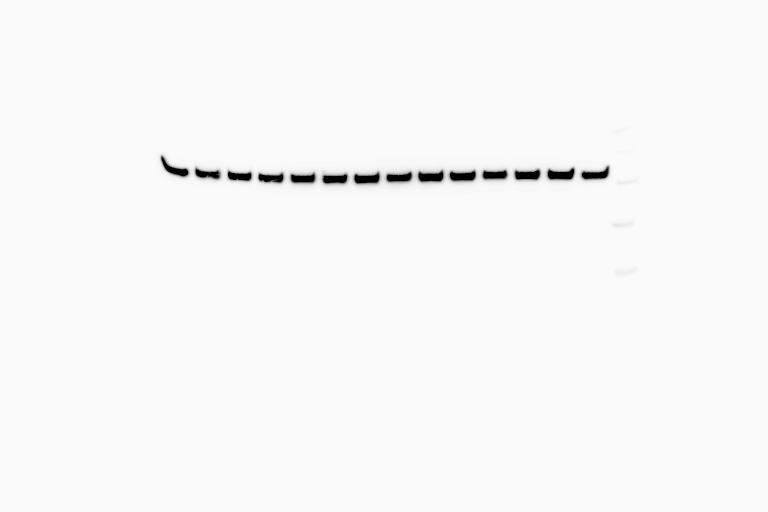

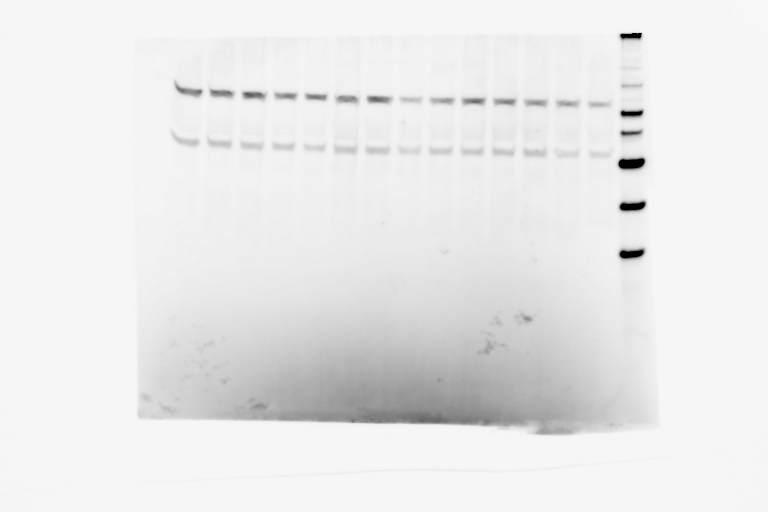


ADH1_Nacc_membrane 1


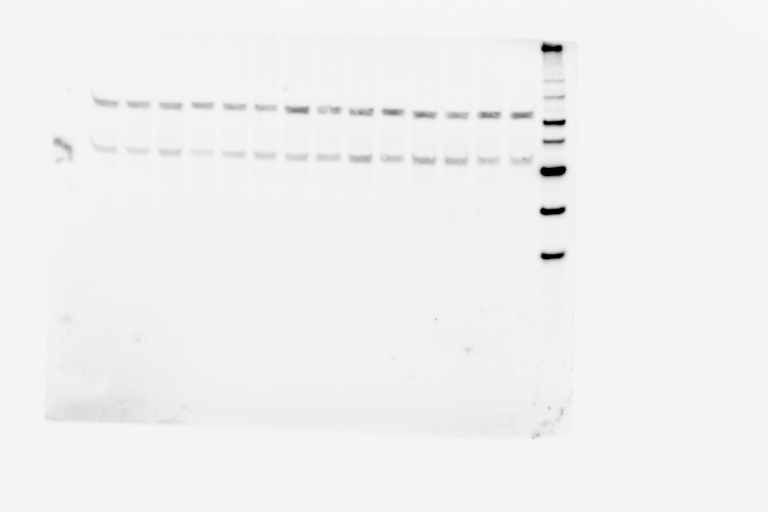


ADH1_Nacc_membrane 2


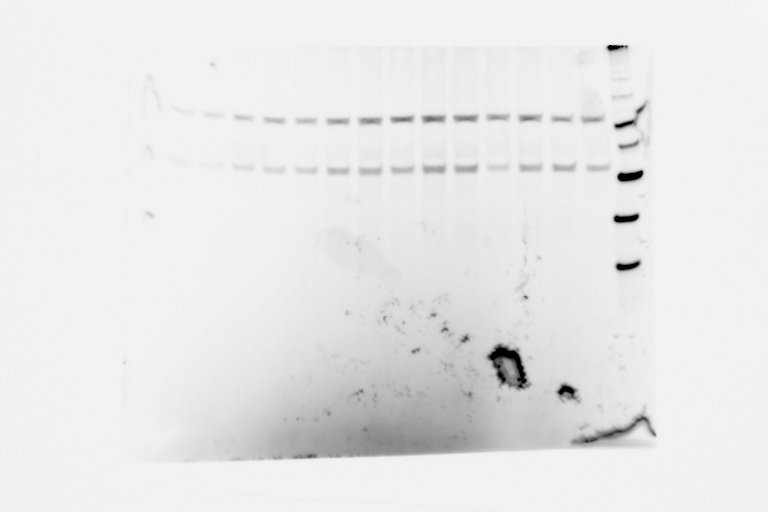

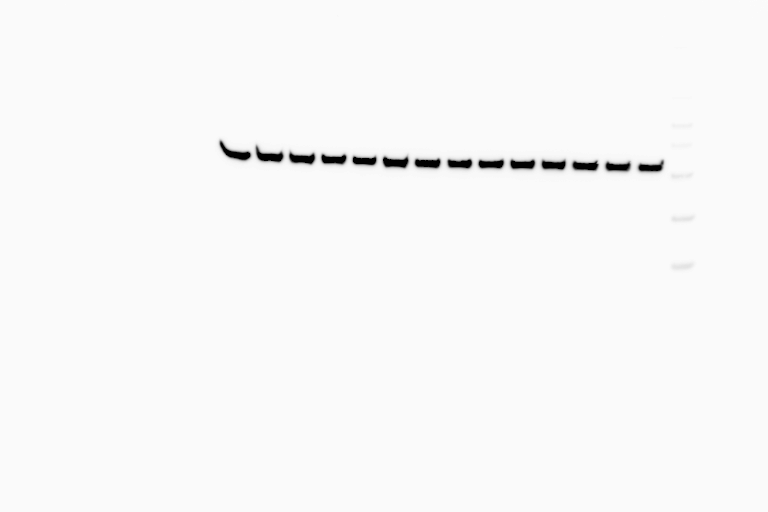


βactin

βactin

βactin

ADH1_Nacc_membrane 3


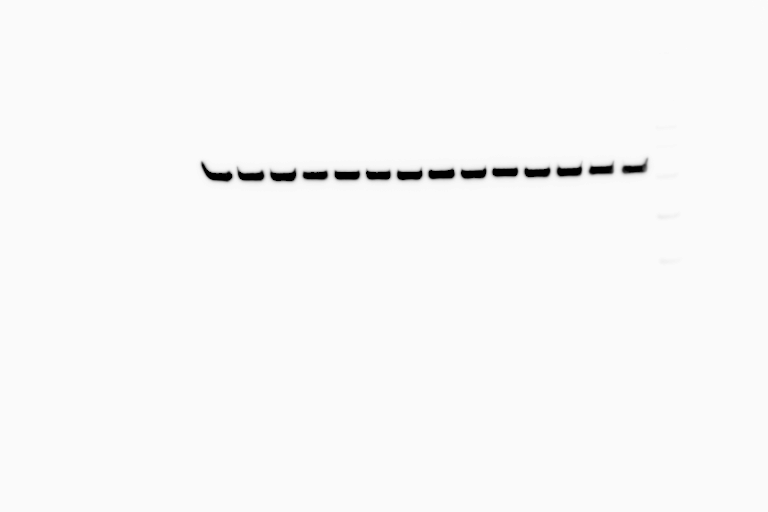


**ADH1_Nacc**
